# Bottom-up Coarse-Graining: Principles and Perspectives

**DOI:** 10.1021/acs.jctc.2c00643

**Published:** 2022-09-07

**Authors:** Jaehyeok Jin, Alexander J. Pak, Aleksander E. P. Durumeric, Timothy D. Loose, Gregory A. Voth

**Affiliations:** Department of Chemistry, Chicago Center for Theoretical Chemistry, Institute for Biophysical Dynamics, and James Franck Institute, The University of Chicago, Chicago, Illinois 60637, United States

## Abstract

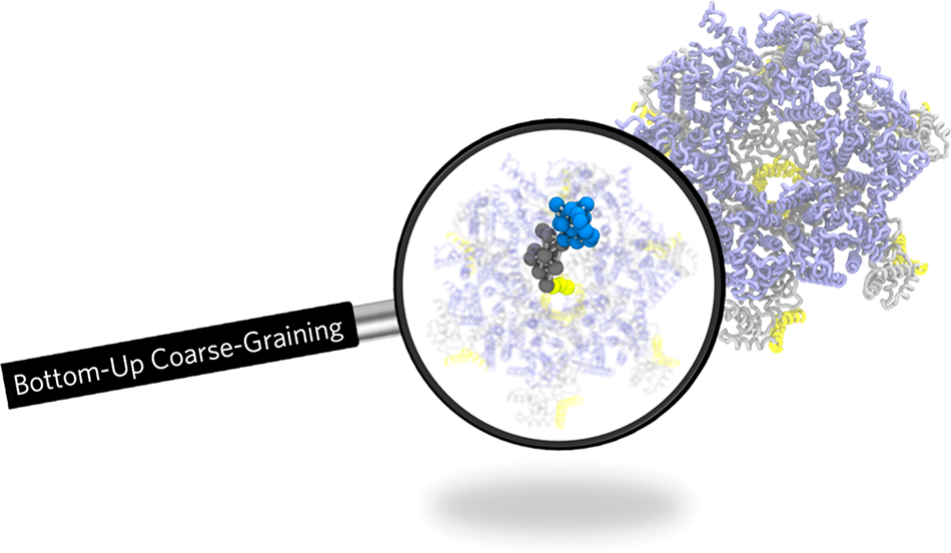

Large-scale computational molecular models provide scientists a means to investigate the effect of microscopic details on emergent mesoscopic behavior. Elucidating the relationship between variations on the molecular scale and macroscopic observable properties facilitates an understanding of the molecular interactions driving the properties of real world materials and complex systems (e.g., those found in biology, chemistry, and materials science). As a result, discovering an explicit, systematic connection between microscopic nature and emergent mesoscopic behavior is a fundamental goal for this type of investigation. The molecular forces critical to driving the behavior of complex heterogeneous systems are often unclear. More problematically, simulations of representative model systems are often prohibitively expensive from both spatial and temporal perspectives, impeding straightforward investigations over possible hypotheses characterizing molecular behavior. While the reduction in resolution of a study, such as moving from an atomistic simulation to that of the resolution of large coarse-grained (CG) groups of atoms, can partially ameliorate the cost of individual simulations, the relationship between the proposed microscopic details and this intermediate resolution is nontrivial and presents new obstacles to study. Small portions of these complex systems can be realistically simulated. Alone, these smaller simulations likely do not provide insight into collectively emergent behavior. However, by proposing that the driving forces in both smaller and larger systems (containing many related copies of the smaller system) have an explicit connection, systematic bottom-up CG techniques can be used to transfer CG hypotheses discovered using a smaller scale system to a larger system of primary interest. The proposed connection between different CG systems is prescribed by (i) the CG representation (mapping) and (ii) the functional form and parameters used to represent the CG energetics, which approximate potentials of mean force (PMFs). As a result, the design of CG methods that facilitate a variety of physically relevant representations, approximations, and force fields is critical to moving the frontier of systematic CG forward. Crucially, the proposed connection between the system used for parametrization and the system of interest is orthogonal to the optimization used to approximate the potential of mean force present in all systematic CG methods. The empirical efficacy of machine learning techniques on a variety of tasks provides strong motivation to consider these approaches for approximating the PMF and analyzing these approximations.

## 1 Introduction

Understanding how molecular phenomena translate into emergent mesoscopic and macroscopic behavior is a common theme throughout biology, chemistry, physics, materials science, and engineering. Experimental techniques have offered microscopic insights into systems from these fields. For example, ensemble-averaged atomic structures can be resolved at high-resolution using X-ray crystallography or cryo-electron microscopy.^1,2^ Alternatively, fluorescence techniques^3,4^ or nuclear magnetic resonance (NMR) spectroscopy^5,6^ can provide dynamic information, albeit at lower spatial resolution. To complement these experimental approaches, theorists leverage classical molecular dynamics (MD) simulations to investigate dynamical phenomena at high spatial resolution, most commonly at the atomistic level.^7^ However, within the space of MD simulation techniques, coarse-grained (CG) modeling and simulation are particularly attractive for the study of systems with hierarchical length and time scales such as biomolecular systems^8−14^ (including UNRES,^15−18^ OPEP,^13,19^ PRIMO,^20^ SIRAH,^21,22^ MARTINI,^23−27^ MS-CG,^28−34^ and REM^35−41^).

By design, CG models are reduced representations of fine-grained (FG) atomistic resolution molecules, where CG sites represent groups of corresponding FG atoms through a process that can be called *mapping*. Effective interactions between the CG sites are *parametrized* to retain the essential aspects of the system of interest under the chosen equations of motion. However, defining these essential aspects depends upon the scientific question at hand and the reference data available, variations in which have led to the development of the *top-down* and *bottom-up* approaches, which are the two general classes of CG models that we will discuss later. CG simulations have three primary benefits compared to FG simulations. First, these models enable simulations of larger systems at appropriate length scales by virtue of the reduced number of particles. Second, a larger integration time step can be used in CG simulations since the removal of highly fluctuating short wavelength atomistic degrees of freedom results in a smoother CG free energy surface that accelerates the sampling under Hamiltonian mechanics. Finally, the construction of useful CG models grants tacit insight into molecular features (i.e., from CG mappings) and energetics (i.e., from CG interactions and associated equations of motion) that are essential for understanding mesoscopic and macroscopic behavior. For these reasons, CG simulations provide perspectives that would otherwise be inaccessible from more detailed atomistic MD simulations, which has driven their continued use and development.

The top-down strategy is perhaps the more typical CG modeling approach. Often, the scientific question posed by top-down CG studies is to determine if a particular set of interactions is capable of reproducing specific macroscopic properties. For example, the original MARTINI CG model for lipids was parametrized to recapitulate partition coefficients.^23−27^ Studies of self-assembling systems have also benefited from top-down approaches. Using simplified geometries and interactions, it has been possible to broadly explore how morphologies may be dictated by a few adjustable parameters.^42−45^ However, as these approaches neglect the direct validation of microscopic details by design, it is unclear if the resultant CG models faithfully reproduce microscopic physics. For example, the original MARTINI model, by construction, lacks a rigorous CG mapping from atomistic degrees of freedom by design^46^ and also may not reflect the underlying nature of atomistically mapped interactions onto the CG representation, such as the correct enthalpy–entropy decomposition for certain calculated potentials of mean force (PMF).^47,48^

Bottom-up approaches use the opposite strategy and attempt to reproduce microscopic (mapped atomistic) statistics. The underlying principles of most bottom-up approaches are that properties observed in reference simulations are to be captured by the correct CG equations of motion describing equilibrium and certain nonequilibrium processes. For example, static properties are to be reproduced by the effective CG interactions as determined by equilibrium statistical mechanical principles. The majority of bottom-up CG approaches aim to reproduce static correlations. One common strategy, which we refer to as *thermodynamic consistency*, is to systematically parametrize CG models such that the sampled distribution recapitulates the multidimensional configurational distribution of their FG counterparts when mapped to the CG phase space.^28,31−33^ Under this criterion, the ideal effective CG Hamiltonians are the conditioned (or CG mapped) many-body PMFs expressed in the CG coordinates or configurations. Reproducing the many-body PMFs using an arbitrarily complex set of functions or “basis set”,^34^ however, is challenging and sometimes problematic, as even if it is computationally feasible to capture the properties of the simulation being analyzed for parametrization, the resulting potential must also describe the larger system of primary interest to the study at hand—an extrapolative task that becomes increasingly difficult as the basis set grows in complexity. Instead, bottom-up studies have explored if CG models that recapitulate reduced sets of microscopic statistics using similarly simplified basis functions are also capable of collectively recapitulating mesoscopic and macroscopic behavior.^9,49−51^ Unlike static properties, dynamical processes are correlated with both temporal and spatial variables and thus can be difficult to represent at CG resolution. Extracting the time evolution of CG systems from the FG reference provides a strategy for rigorously integrating the many-body nature of time-dependent processes into CG models.^52−54^

Bottom-up CG models are created to generate samples that systematically approximate high-dimensional data produced by a reference model. This approach is fundamentally similar to those of contemporary methods in machine learning (ML).^55,56^ Algorithms for high-dimensional regression have been applied as the building blocks for force fields that recapitulate high order correlations in CG mapped atomistic data.^57−60^ Simultaneously, ML techniques focused on directly generating samples from high-dimensional distributions have provided approaches for quantifying the error in existing CG models and more efficient methods for generating atomistic configurations. These novel generative approaches have additionally shown promise in producing atomistic configurations from CG simulations (an approach often referred to as “backmapping”).^61−69^

In this Review, we summarize recent advances in bottom-up CG modeling (with some, albeit abbreviated, historical context) and discuss promising future directions. In particular, we focus on how bottom-up CG models can be derived by establishing a systematic connection between the microscopic (atomistic) and the reduced descriptions. We first review fundamental concepts in CG models that have been proposed over the past two decades. We then discuss limitations and challenges in CG modeling in terms of consistency, representability, and transferability. We briefly survey recent scientific findings and breakthroughs that benefited from methodological advances, e.g., concepts from ML. We conclude with a brief overarching summary and future outlook toward the next generation of bottom-up CG modeling.

## 2 Basics of Bottom-Up Coarse-Grained Modeling

Two ingredients are necessary for any bottom-up CG modeling recipe. While intertwined, these processes are typically performed separately. First, one needs to define the CG mapping that formally defines the correspondence between the FG and reduced resolutions. Then, once a mapping is selected, the CG mechanics need to be defined in the desired CG phase space on the basis of the FG statistics mapped onto that space. Various techniques can be applied for parametrizing the interactions governing the CG equations of state. These two ingredients are not only essential to construct CG models but also to provide the theoretical basis to understand the challenges underlying bottom-up CG modeling, i.e., consistency, transferability, and representability. Below, we briefly describe these essential steps required for bottom-up CG modeling and discuss how they are related to the aforementioned challenges.

### 2-1 Coarse-Grained Mapping

The process of applying a CG mapping reduces the high-dimensional atomistic phase space to a low-dimensional CG phase space. Ideally, this should involve mapping over both configurational and momentum variables in phase space, but most molecular CG models do not involve momentum in their equilibrium distribution consistency, and under this assumption, the majority of CG mappings can be readily applied to only the configurational variables. The field of chemistry has an established history of breaking complex molecules into moieties and functional groups in order to predict and understand atomistic behavior. This process provides an intuitive basis for designing CG configurational mappings. Even though there are numerous mapping schemes that one can take in order to preserve the desired behavior in CG models, the resultant CG interaction associated with the specific mapping should be designed based on the statistical mechanical principles given by the CG methodology, which will be reviewed in Section 2-2 below.

#### 2-1.A Based on Real Particles

Representing each chemical moiety as a group of CG sites is perhaps most easily realized by defining each CG site as a weighted average of the configurations of various atoms. For these real particle-based mappings, the CG mapping operator on configurational variables is expressed as a set of *N* linear functions **M**(**r**):(**M**_1_(**r**),...,**M**_*N*_(**r**)), where the FG coordinates can be mapped into CG site *I* following
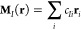
1

One example is the center-of-mass mapping (*c*_*Ii*_ ∝ *m*_*i*_), enabling one to retain important molecular configurations and momenta at the reduced level. For force-based CG methodologies, the center-of-mass mapping provides thermodynamically consistent forces that act on the center-of-mass phase space variables in comparison to atomistic forces.^32^ However, information beyond configurations may be lost using the center-of-mass mapping. Recent advances have suggested that one can alternatively perform center-of-charge mapping, which is a reweighted mass based on the partial charges of atoms within CG sites,^70^ to better encode electrostatic information^71,72^ for systems in which electrostatic interactions play a major role, e.g., ionic liquids.^73^ It is also conceivable that the geometry of the system can be better conserved by performing center-of-geometry mapping as *c*_*Ii*_ = 1/*n*_*I*_, where *n*_*I*_ denotes the number of FG particles involved in CG site *I*.

#### 2-1.B Based on Virtual Particles

Interactions that are centered on CG sites mapped from the FG variables alone can be a limitation in CG modeling. Particles that do not explicitly represent specific FG particles can be included as additional interaction centers, thereby introducing a general means to increase the expressivity of the desired model; we holistically refer to these particles as “virtual sites”. Virtual sites have been used to impart subtle anisotropic projections of forces acting upon real sites. One prototypical example of this idea is the atomistic TIP4P water model.^74^ Similar types of virtual sites have been used in the context of high-resolution CG models, notably for sterols and for aromatic hydrocarbons.^75,76^ Overall, virtual sites can be thought of as relatively inexpensive augmentations to conventional real particle-based mappings.

Virtual sites in CG models have been increasingly utilized in recent years, most predominantly in top-down CG models.^77−79^ Moreover, virtual CG sites were required to describe directional interactions at protein–protein interfaces that are responsible for viral self-assembly.^44,80−86^ However, the use of virtual sites in bottom-up CG models has been limited due to a lack of systematic rules that describe effective virtual site interactions. One proposed approach is to use the so-called center-of-symmetry framework that maintains thermodynamic consistency while preserving the molecular asymmetry via virtual particles.^76^ We note that the necessity of preserving molecular symmetry in CG mapping is still an open problem,^87^ but the center-of-symmetry framework can effectively encode the missing quadrupole information into CG models (e.g., π–π stacking from benzene rings), enhancing the fidelity of structural correlations and transferability. These early successes demonstrate the utility of virtual sites and motivate the need for additional efforts to determine systematic rules for virtual site mappings and effective interactions for complex molecular systems, e.g., polymers.^88^ Another approach that shares a similar physical principle is to introduce virtual sites that help to represent the effects of explicit solvent in implicit solvent models.^89^ These so-called “solvent-free” CG models can potentially provide an accurate and transferable CG modeling for biomolecules, e.g., amphiphilic assemblies of lipids.

#### 2-1.C Mesoscopic Mapping: Clustering

On larger mesoscopic scales, CG particles can instead be represented as supramolecular “blobs”, and the CG mapping at this resolution becomes less clear than at the molecular level. If the target system is composed of bonded systems, one can still employ a linear center-of-mass mapping.^90−93^ However, the same strategy cannot be applied for unbonded systems since the construction of CG blobs becomes a nonlinear and time-dependent procedure. As developed by Español and co-workers, who introduced the Voronoi cell representation,^94^ suitable mesoscopic representations for unbonded systems must be obtained via alternate approaches such as clustering methods other than conventional center-of-mass and related mappings. One such example is the application of the *k-*means clustering algorithm to unbonded fluids,^95,96^ and another option proposed by Praprotnik et al.^97,98^ uses spatial tessellation at adaptive resolutions. By adjusting the center of each Voronoi cell based on its center-of-mass, these clustering methods allow for mesoscopic CG blobs to faithfully represent their FG counterparts. Yet, conformation-based clustering suffers from the nonanalytical nature of the mapping process, which makes the derivation of CG interactions impractical, and requires frequent reclustering over the simulation. This limits the development of highly CG models to study mesoscopic behavior.^99^ Hence, several alternatives have been reported in the literature, including a spherical CG blob mapping by Ayton et al.^100^ This latter work was also extended (mainly, but not completely, in a top-down manner) to treat biomolecular membranes, with and without bound proteins.^61,101−104^

In general, the nonlinear, nonanalytic, and iterative nature of the mesoscopic CG mapping is considered a major bottleneck in mesoscale modeling of unbonded molecules (i.e., liquids). Recently, a “dynamic mapping” scheme was developed by mapping velocities instead of configurations, which only requires the initial configuration from smoothed centroidal Voronoi tessellations.^105^ To note, this mapping scheme is based on a Lagrangian description to track individual fluid particles, but a complementary Eulerian description can also be established in a similar vein.^106^ In turn, this new approach allows stable propagation of the CG blobs over time, indicating its applicability to various fluids,^107^ e.g., heterogeneous multiphase systems.

#### 2-1.D Backmapping

Due to information loss during the coarse-graining process, there is a seemingly inherent lower bound to the CG resolution when creating a model for a particular scientific question. While the CG resolution can be tuned to optimize the level of detail remaining as to only include the relevant parts of the system at hand (see Item E later), this resolution may itself prove to be too computationally expensive. As a result, it is often desirable to recapture the full FG details by “backmapping” the CG configurations onto FG configurations.^108^ In prior studies, backmapped structures from CG simulations have been used as starting points for FG simulations in order to improve FG sampling.^61−69^ Generally, the procedure is performed in two steps. First, an initial model for the backmapped FG structure is predicted using geometric algorithms. Then, the structure is equilibrated using a short MD simulation. However, due to the degeneracy associated with the CG mapping, this process is highly nontrivial, and some recent advances have introduced ML techniques to perform backmapping (see Section 6). Early work also developed reasonably rigorous statistical mechanical methods for backmapping from the CG resolution to obtain the Boltzmann distribution in the FG variables (or close to it).^109−113^

#### 2-1.E Optimal Resolution

Along with CG mapping, the resolution of CG models impacts the resultant CG model phase space and the performance of CG models in reproducing the values of key observables. These factors are particularly important for modeling complex biomolecules, where grouping different atomistic entities becomes less clear. While there is no definitive answer to this problem, the optimal resolution for the desired CG models can be chosen to maximally reproduce the key observables from CG models or the loss of information from the coarse-graining process.^114^

Notably, essential dynamics coarse-graining (ED-CG) has been developed to systematically estimate the optimal partitioning scheme for large biomolecules by recapitulating key dynamics (or essential dynamics).^115^ ED-CG can be employed in conjunction with principal component analysis (PCA) of atomistic trajectories^115^ or CG models of large proteins^116^ described by an heterogeneous elastic network model (HeteroENM).^117^ Various determination protocols have also been suggested recently to determine the optimal CG representation, e.g., constrained minimization,^118^ stepwise optimization,^119^ and fluctuation maximization.^120^

On the other hand, an alternative approach can be achieved by minimizing the loss of information accompanied by the coarse-graining process. References (121 and 122) systematically investigated this concept by reproducing underlying fluctuations in terms of the CG model spectrum using a Gaussian network model, which can be further extended to elastic network models (ENMs).^123^ As this information loss is intrinsically related to the mapping entropy from the coarse-graining process, ref (124) suggests a CG mapping optimization strategy by minimizing the mapping entropy with the aid of ML techniques^125^ or enhanced sampling algorithms^126^ (e.g., Wang–Landau^127,128^) in a way to preserve the maximum possible FG information. We emphasize that the mapping entropy introduced here is one of the most central quantities not only to determine the CG mapping but also to correctly understand the representability and transferability of CG models as will be discussed in Section 2-4.

Beyond a single resolution, CG models with multiple resolutions can be a way to capture different levels of detail in inhomogeneous systems. However, these so-called adaptive resolution models also require a concurrent coupling among multiple levels of resolution and changing the particles’ resolution on the fly. One of the most commonly practiced frameworks, the adaptive resolution simulation (AdResS) approach, allows for a smooth transition between different resolutions by introducing a hybrid transition region.^129^ Then, the coupling between each resolution is designed based on the ensemble of interest and desired resolution ranging from quantum to hydrodynamics levels. For the molecular level, the original AdResS used a force coupling,^129^ and then Hamiltonian AdResS (H-AdResS) was developed by designing the global Hamiltonian.^130−132^ For different spatial levels, several AdResS-based schemes have been developed, such as for larger proteins,^133^ continuum hydrodynamics,^134,135^ systems with quantized nuclei,^136,137^ open systems,^138,139^ and the grand canonical ensemble.^140,141^ One particularly nontrivial question would be to determine the thermodynamically consistent bottom-up interactions at the multiple resolutions. We will briefly discuss this in Section 2-4.

### 2-2 Coarse-Grained Mechanics

**Figure 1:**
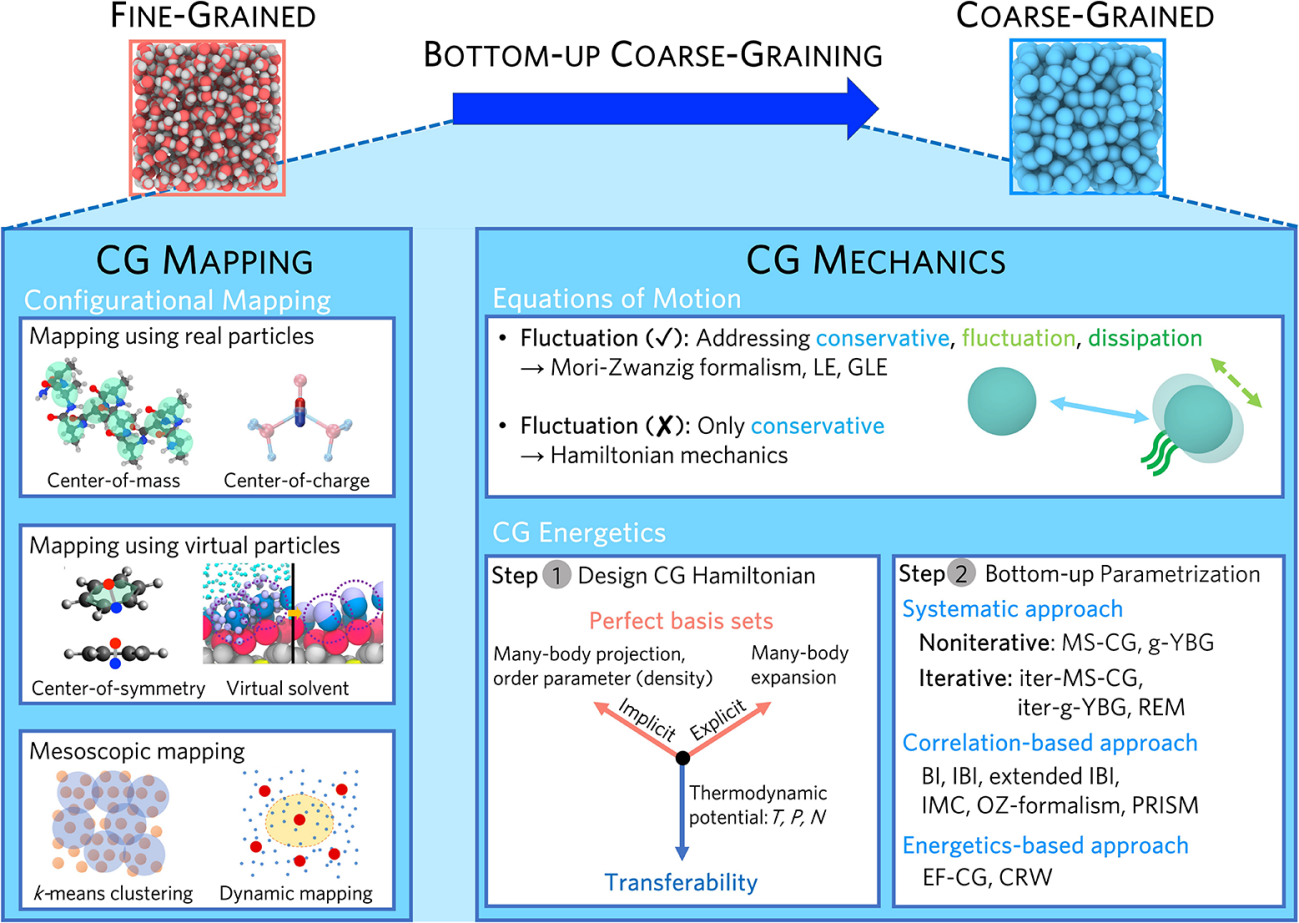
Broad summary of bottom-up CG modeling. Based on FG reference statistics, CG modeling is composed of two steps. (1) CG mapping (often performed on configurational variables) involves real or virtual CG particles at molecular resolution or includes mesoscopic mapping at coarser resolutions. (2) CG mechanics are defined by the specific consistency criteria and design principles that determine the CG equation of motion and CG interactions. CG equations of motions are generally chosen based on the target dynamical information, e.g., with or without fluctuation forces. CG interactions are determined by the designed CG Hamiltonian, which may suffer from an imperfect basis set and transferability issues. Bottom-up parametrization methodologies are then applied to yield effective CG interactions that optimally approximate the level of physics specified by the consistency criteria and design principles.

Once the mapping operator is decided, CG mechanics must be determined via (1) the equations of motion for the CG phase space variables and (2) the effective CG interactions from the chosen equation of motion in phase space, which will then enable CG simulations to be performed. Similar to the process of CG mapping, in the canonical (constant *NVT*) ensemble, the effective CG interactions are only dependent on the CG configurations. The essential building blocks for CG models are summarized in Figure 1.

#### 2-2.A Coarse-Grained Equations of Motion

CG equations of motion and energetics affect the fidelity of CG models in different ways. In order to correctly address dynamical properties, CG models should faithfully represent the friction and fluctuations observed in the reference FG system.^52−54^ On the other hand, the equilibrium static properties, e.g., structural correlations, are not dependent on dynamical behavior. Correct recapitulation of static correlations is possible through only conservative interactions. While the latter can be propagated under Hamiltonian mechanics, the proper dynamics (time-dependent behavior) requires equations of motion accounting for nonconservative interactions, e.g., the generalized Langevin equation. In this section, we specifically discuss the performance of CG models in terms of static correlations. The related discussion on dynamical properties is presented in Section 5.

#### 2-2.B Coarse-Grained Energetics: Design Principles

In general, it is impractical to determine exact forms of the renormalized many-body CG interactions. Therefore, constructing approximate bottom-up CG models is performed in two sequential steps. The first step is to design the form of the CG Hamiltonian in terms of configurational variables. This is often done by adopting molecular mechanics functional forms similar to that of the atomistic description, such that the approximate CG interactions are written as a combination of analytical and tractable forms, including bond, angle, torsion, and pair nonbonded interactions. Various design principles for the CG Hamiltonian will be described in detail in Section 3. The second step is to determine the interaction parameters for the defined CG Hamiltonian, which is the focus of this subsection.

### 2-3 Coarse-Grained Force Fields

#### 2-3.A Bottom-up Philosophies

In order to address a myriad of chemical and biological systems, most CG methodologies provide a general principle to determine CG interaction parameters regardless of the CG Hamiltonian form. This is often manifested in the bottom-up manner by enforcing certain statistical mechanical principles to maintain the fundamental properties of the FG system. Depending on the microscopic target of interest (thermodynamic properties or static correlations), various bottom-up CG methodologies have been proposed in the field. In this subsection, we briefly survey some of the leading strategies for approximating CG energetics, and we will review naturally emerging issues in Section 3.

#### 2-3.B Based on Variational Principles

As noticed from the CG equations of motion, forces are central to equilibrium thermodynamics, and several methodologies have been designed based on the conservative forces. The idea of employing force-matching (without coarse-graining) originated from the early work of Ercolessi and Adams^142^ and from Izvekov, Parrinello, Burnham, and Voth^143^ to define molecular mechanics force fields on an *ad hoc* basis from *ab initio* calculations. The extension of force-matching to CG configurational space was established with the Multiscale Coarse-Graining (MS-CG) method developed first by Izvekov and Voth for biomolecular systems^28,30^ and liquids.^29^ This advance was accomplished through a recognition that force-matching could be carried out along with a resolution reduction (coarse-graining) and that this would be a variational route to determine the many-body PMF for the CG variables. Later, in a series of papers,^31−34,70,144−151^ the MS-CG method was more fully developed and explored. By design, the MS-CG methodology determines the effective force field acting on CG site *I*, **F**_*I*_(**M**(**r**^*n*^)), by minimizing the least-squared force residual χ^2^[**F**] between a target CG model and the FG counterpart. Here, **M** denotes a mapping operator that maps the FG configuration **r**^*n*^ to the CG configuration **R**^*N*^. These force differences are often expressed as a quadratic residual, and thus, a systematic determination is possible by variationally minimizing this force metric
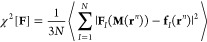
2where **F**_*I*_(**M**(**r**^*n*^)) is the unknown CG forces at the CG configuration, and **f**_*I*_(**r**^*n*^) is the projected microscopic forces on the CG site *I.* The unknown CG forces can be linearly expressed through the CG force field parameters  =  via **F**_*I*_(**R**^*N*^) ***= –***∇_*I*_*U*_CG_(**R**^*N*^). In general, a two-body (pairwise) approximation is often adopted to express the CG force field basis sets, and subsequent algorithmic advances^34,152^ introduced spline interpolation to describe force field parameters as  =  =  which reduces the least-squares problem in eq 2 to an overdetermined system of linear equations,^153^ resulting in the following matrix equation
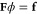
3

In eq 3, **F** is the force matrix calculated from the CG configurations and the CG force field parameters ϕ, and the column vector **f** represents the projected FG forces at the CG resolution. From a statistical mechanical perspective, the MS-CG methodology satisfies the thermodynamic consistency between the FG and CG phase spaces.^32,33^ It is important to note that this approach to training a bottom-up CG force field from FG data can be considered an early example of “machine learning”, which has become very popular in recent times (including for coarse-graining), albeit a deep neural network^59^ was not utilized in that early MS-CG work of almost 20 years ago.

In contrast to the force-based metric, Shell and co-workers have identified and implemented the information-theoretic relative entropy as a target metric.^35−41^ Relative entropy is defined as the differences between the FG and CG probability distributions, given by the Kullback–Leibler divergence^154^
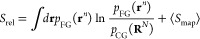
4where ⟨*S*_map_⟩_CG_ denotes the mapping entropy (introduced in Section 2-1) defined by a mapping operator, *S*_map_ = ln ∫δ[**M**(**r**) **– R**]*d***r**. Thus, minimizing *S*_rel_ enforces minimizing the log difference between the FG and CG probability distributions. Based on this metric, relative entropy minimization (REM) can be expressed within the canonical ensemble as *S*_rel_ = β⟨*U*_FG_ – *U*_CG_⟩_CG_ – (*A*_FG_ – *A*_CG_) + ⟨*S*_map_⟩, where *U* and *A* denote the internal energy and free energy, respectively. Then, a set of CG model parameters {*λ*_*i*_} can be variationally determined by minimizing the relative entropy differences between the FG and CG systems, resulting in the following conditions for local optimality^38^
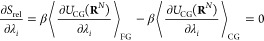
5a
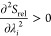
5b

Due to the systematic nature of REM, several variations were reported to enhance the predictability of CG models.^155^ The connection between MS-CG and REM has also been analyzed:^38,156^ both will give the exact many-body CG variable PMF if a “perfect” basis set is used to describe the CG interactions, but the two approaches will differ in complementary ways if more approximate basis sets are used.

Even though the aforementioned approaches can variationally determine the effective CG interaction parameters, it is not immediately clear if the resultant CG models will reproduce a particular target atomistic correlation correctly given the approximate nature of the CG Hamiltonian as well as the basis set chosen to describe the CG interactions. Recent studies have, however, shed light on such connections. Noid et al. demonstrated that the MS-CG method determined from eq 2 satisfies the Yvon−Born–Green (YBG) hierarchical equation,^157^ indicating that MS-CG with two-body interactions attempts to capture two-body and three-body structural correlations.^31^ In a related fashion, Chaimovich and Shell showed that REM guarantees capturing any *n*-body statistical correlations that are explicitly represented in a corresponding *n*-body CG Hamiltonian.^38^

By establishing links between force-based models and structural correlations, explicit consideration of FG structural correlations can result in modified strategies for force field parametrization. Notably, Mullinax and Noid provided a practical link by developing the generalized YBG (g-YBG) framework to determine optimal interaction potentials for complex classical force fields by utilizing only structural correlation functions.^158,159^ The g-YBG approach was readily applied to CG systems on the basis of the MS-CG framework, where the structural correlations were used instead of forces.^158−162^ This observation is based on eq 3 that the normal equation form can be constructed by acting a transpose **F**^T^ on the left-hand side. This produces the following equation
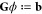
6where **G** ≔ **F**^T^**F** and **b** ≔ **F**^T^**f**. In the g-YBG approach, *b* is expressed in terms of a set of structural correlation functions,^158−160^ and **G** contains the ensemble average of cross-correlations between the CG degrees of freedom.

An iterative refinement to eq 6 can improve the reproduction of specified correlation functions. Namely, two iterative schemes are possible. The first approach is to update the *G* matrix iteratively by matching the CG forces at a given CG structure to the mapped FG forces. Cho and Chu applied this scheme to the MS-CG methodology,^163^ and Rudzinski and Noid made similar extensions to the g-YBG framework.^164^ Another iterative treatment is to recalculate the *b* column vector by matching the CG forces at a given FG structure to the CG forces at a given CG structure. The latter approach is equivalent to matching expectations of basis function derivatives, as demonstrated by Lu et al.^165^ Another recent advance has resulted in a noniterative parametrization scheme, while still based on eq 6, in order to directly reproduce pair correlations by transforming the atomistic cross-correlations.^166^ Lastly, the parametrization strategies discussed above can be improved by leveraging ML techniques. While general principles and examples of ML-based CG parametrization will be discussed in Section 6, we briefly note here that force-matching and relative entropy ideas can be translated into ML. For example, the Kullback–Leibler divergence in eq 4 can be extended to general *f-*divergence,^58^ and the force-matching scheme in eq 2 can be utilized to train the CG free energy functional.^59^ Beyond force-matching, the effective *flow* by combining eq 2 and 4 can also be trained to recapitulate the CG probability density.^167^

#### 2-3.C Based on Static Correlations

Alternatively, several bottom-up CG approaches have been specifically designed to capture target static correlations (as opposed to the many-body PMF of the CG variables like MS-CG and REM). One of the earliest attempts was to capture pair correlations, or radial distribution functions (RDFs), from FG systems^168,169^ based on Henderson’s uniqueness theorem,^170^ which asserts that there is a unique pair potential that gives rise to a given RDF. Under dilute (low-density) conditions, one can ignore the many-body correlations in the system, and the effective pair interactions can be approximated based on the RDF or *g*_FG_(*R*). This approach is known as the (direct) Boltzmann inversion^171^
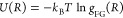
7

However, most condensed matter systems are not at the low-density limit, and thus, the model RDF from the CG trajectory using eq 7 often deviates from the reference RDF (*g*_FG_(*R*)). This deviation can be corrected using an iterative scheme, known as iterative Boltzmann inversion (IBI), where one can iteratively improve the fidelity of the CG models by updating the CG interactions according to
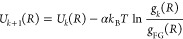
8with a convergence rate α.^172^ Even though the IBI approach has been applied to various chemical systems with its relatively simple update scheme, eq 8 is neither a variational approach nor strictly based on rigorous statistical mechanical principles, and thus, improving IBI models to resolve thermodynamic issues cannot be done systematically. As a result, several IBI-based approaches require a heuristic, phenomenological theory to design the parametrization strategy. We note that ref (173) substantiates the well-posedness of IBI under certain conditions, but not all systems of interest fall into this case. Also, pair correlation-based CG methodologies suffer from numerical degeneracy in the RDF. Even though Henderson’s theorem proves uniqueness, several practically constructed CG models (such as those parametrized via IBI and REM) can yield similar RDFs due to the sensitivity of pair distributions as well as numerical issues. The pair distribution itself intrinsically contains configurational degeneracy,^174^ and recent work by Wang et al. systematically demonstrated that the RDF can be insensitive to pair interactions,^175^ as substantiated by previously reported systems using various CG modeling methods.^176−178^ Therefore, several extensions from IBI have been designed to surmount these issues, including the multistate generalization (multistate IBI) by McCabe and co-workers^179,180^ as well as thermodynamic property-based IBI approaches such as Kirkwood−Buff IBI by van der Vegt and co-workers^181^ and −IBI by Junghans and Mukherji.^182^

Similarly, an approach named inverse Monte Carlo (IMC) by Lyubartsev and Laaksonen also targets the reference RDF and determines the CG interactions in an iterative manner.^183,184^ Yet, these IMC iterations often suffer from computational overhead due to the sampling of all possible configurations.^185^ In light of these issues, recent methodologies have been designed based on an integral equation approach that can approximate the many-body correlations via closure equations. For example, Guenza and co-workers have proposed an Ornstein–Zernike integral equation-based analytical approach to determine the effective CG interactions for polymeric systems.^186−188^ It is also possible to utilize an inverted integral equation as an initial guess for IBI to improve parametrization efficiency.^189^

#### 2-3.D Based on Energetics

As opposed to target- or correlation-based approaches, one can also directly derive CG interactions from reference FG systems. Based on atomistic energetics, this class of approaches attempts to extract the reduced energetics, i.e., forces or free energies, directly from the atomistic energetics. For example, by borrowing from the force-matching philosophy, the effective force CG (EF-CG) approach computes the averaged forces acting on the groups of atoms by projecting onto corresponding radial vectors.^190^ By directly extracting the force information from the atomistic simulations, the CG Hamiltonian is approximated as atomistically averaged forces, and EF-CG can be derived from an averaged description of the MS-CG methodology. Similar averaging schemes, as well as the EF-CG method, can be employed to capture structural correlations,^191−194^ since these averaged interactions correctly account for the existing pair- and many-body correlations in FG systems. Similarly, in order to correctly capture the underlying FG energetics, a recent extension of the MS-CG method^195,196^ (called “energy-matching”) has shown that one could variationally determine the pair energy functions by minimizing the energy differences akin to eq 2. This allows for the pinpointing of energetic contributions from the many-body CG variable PMF, which is advantageous for better understanding of transferability.

A direct assessment of the PMF is also possible by explicitly computing effective pair potentials following the definition of the PMF in the low-density limit. In this case, by adopting a free energy perturbation approach, effective CG interactions can be computed via conditional reversible work (CRW).^197^ This fragment-based CG approach can be advantageous in terms of transferability for different system conditions, yet, by design, it does not guarantee that the static correlations will be correctly addressed.^198−202^

### 2-4 Representability and Transferability in Coarse-Grained Modeling

#### 2-4.A Accuracy in Coarse-Grained Modeling

The accuracy of bottom-up CG models is undoubtedly important as it reflects the fidelity of CG models relative to the FG reference. Yet, appropriate metrics for accuracy are ambiguous at present. In this section, we define three separate yet related measures of bottom-up CG model fidelity: *consistency*, *representability*, and *transferability*. The use and importance of each of these measures are dependent upon the scientific question of interest. It is therefore worthwhile to discuss each of these metrics with the understanding that all three contribute to the overall accuracy of a bottom-up CG model. It should be noted that to an extent top-down CG models do not satisfy some or all of the properties discussed below, and it will generally mean that those top-down models are not consistent with statistical mechanics (meaning they do not provide a direct connection between the FG and CG worlds but are instead primarily models in the larger sense of the word).

#### 2-4.B Consistency

Consistency refers to the specific statistical mechanical principle used to generate a bottom-up CG model from a reference FG model. This consistency can serve as the theoretical basis to derive CG force fields or be utilized as the criterion to evaluate how the constructed CG models capture FG statistics in a consistent manner.

Since the emergence of CG modeling as a “field”, various consistency conditions have been proposed. For example, the MS-CG methodology was initially built upon the consistency of excess free energies, asserting that the CG model should provide an excess free energy *A*_ex_ = −*k*_B_*T* ln [∫*d***R**^*N*^*e*^–*βU*_CG_(**R**^*N*^)^/*V*^*N*^] that is identical to the FG excess free energy *a*_ex_ = −*k*_B_*T* ln [∫*d***r**^*n*^*e*^–*βu*_FG_(**r**^*n*^)^/*V*^*n*^].^203^ This condition is sufficient to derive the effective CG interaction form in configurational space. However, in order to consider the full phase space, the most commonly used criterion built upon equilibrium statistical mechanics is *thermodynamic consistency*,^31−33^ indicating that the CG variables *p*_CG_(**R**^*N*^, **P**^*N*^) should have exactly identical probability distributions as compared to that of FG variables *p*_FG_(**r**^*n*^, **p**^*n*^) that are mapped to the specific CG phase variables via the mapping operator **M**:(**r**^*n*^, **p**^*n*^) → (**R**^*N*^, **P**^*N*^). Both probability distributions follow Boltzmann sampling at equilibrium, and configurational and momentum variables can be separated as discussed earlier. Mathematically, this can be expressed as
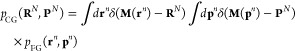
9where the mapping operators on configurational and momentum variables are folded into the delta functions, which are understood to be a product of delta functions in the expressions here, one for each FG to CG mapping, i.e.,  and . This thermodynamic consistency can be reduced into configurational and momentum consistency relationships, respectively
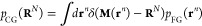
10a
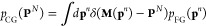
10b

The configurational consistency implies that the effective CG interaction potential should have the following form
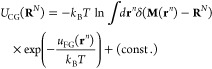
11

Eq 11 also demonstrates that the effective CG interaction that ensures configurational consistency is equivalent to the many-body PMF, which is a configuration-dependent free energy function. Notably, this condition serves as a design principle for CG force fields,^33^ and a rigorous link between eq 11 and force-matching from eqs 2 and 3 was shown in ref (32).

Furthermore, the renormalized nature of the CG interactions shown in eq 11 points to the nontrivial challenge in designing a multiresolution CG model (one of the first systematic attempts can be found in ref (204)). Especially, at the AdResS-level linking the FG and CG resolutions (assuming that we map to *n*_0_ FG particles and *N*_0_ CG particles), the overall renormalized interaction *U*_FG/CG_(**r**^*n*^, **R**^*N*^) should be written as
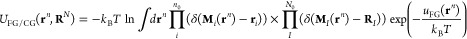
12where the constant term that is independent of configurational variables is omitted for simplicity. In eq 12, the delta functions containing the mapping function (to two different resolutions) enter inside the integrand of the many-dimensional integral. Therefore, the overall multiresolution interaction must be considered to be renormalized at both levels, FG and CG. However, this systematic thermodynamic connection is often missing in many current multiresolution models.^205−207^ For example, the AdResS treats the overall potential as^130^
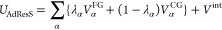
13where coupling parameter *λ*_*α*_ varies from 0 to 1 to couple the FG and CG systems with their bulk (naked) interactions *V*_α_^FG^ and *V*_α_^CG^, respectively. While this interpolative approach has shown to be able to capture various thermodynamic properties at equilibrium, the thermodynamic consistency principle of eq 12 suggests that there is no formal theory to support the idea that eq 12 can be written in terms of the “naked” FG potential as done in eq 13. As such, since the topic of adaptive and multiresolution modeling is a complex one,^204^ it can still benefit from additional development based on rigorous statistical mechanical principles. In the remainder of this Review, we will therefore only focus on CG models with a single level of resolution.

While configurational consistency reveals the many-body nature of CG interactions, momentum consistency imposes a rigorous condition when designing CG mapping operators. Unlike configurational interactions, the momentum contribution to the system Hamiltonian is rather simple if the mapping functions are linear:  and  for the FG and CG systems, respectively. Moreover, this form of the momentum contribution, combined with eq 10b, asserts that no FG particles can be mapped to more than one CG site.^32^

#### 2-4.C Representability

The general connection between microscopic statistics and dynamical or thermodynamic observables (e.g., internal energy, entropy, pressure, temperature, etc.) has been rigorously derived through statistical mechanics. It is these relationships that allow computer simulations of FG models (and generally only FG models) to quantitatively predict experimental properties; in this paper, we holistically refer to these relationships as *representability*. In the case of dynamical representability, we will refer to how well the CG models reproduce FG dynamical properties, and this is often related to the choice of equations of motion used throughout the CG simulations. We specifically discuss dynamical representability in Section 5. Furthermore, in contrast to time-dependent properties and dynamical representability, the static picture of CG models can be assessed via structural representability. Structural representability is defined as how well the CG models reproduce FG structural correlations, which is highly dependent on the quality of the approximated conservative interactions at the CG level. This particular topic will be discussed in Section 3.

Our particular interest in this section is in representability issues that arise from thermodynamic inconsistencies observed in various CG systems, which are broadly referred to as the (thermodynamic) representability problem.^49−51^ Perhaps the most common example is the trade-off between consistent recapitulation of pairwise correlations (via configurational consistency) and dramatic overestimation of pressure as computed from the virial theorem due to missing degrees of freedom in the CG model.^208,209^ From a statistical mechanical perspective, the representability problem is rooted in the nature of the renormalized degrees of freedom. Due to the missing configurational and momentum variables in the CG phase space, the FG observables from the FG ensemble are not always equivalent in value to their CG counterparts if the latter are simply calculated using the same expressions as in the FG model.^49−51,121,195^

To demonstrate this perspective on representability, consider the fact that the ideal effective CG Hamiltonian in the CG configurational space, *U*_CG_(**R**^*N*^), in the canonical ensemble under thermodynamic consistency is equivalent to the many-body PMF.^31−33^ As a projection of the free-energy, *U*_*CG*_ has two important attributes. First, *U*_CG_(**R**^*N*^) is clearly state point-dependent, i.e., *U*_CG_(**R**^*N*^) = *f*(**R**^*N*^, *V*, *T*). Second, *U*_CG_ encodes both energetic (*E*_CG_) and entropic (*S*_CG_) contributions, i.e., *U*_CG_(**R**^*N*^) = *E*_CG_ – *TS*_CG_(**R**^*N*^);^148^ the entropic contribution represents the entropy “lost” and folded into the CG interactions due to CG mapping, that is, the entropy associated with the FG degrees of freedom that map to the same CG configuration. Therefore, evaluating ⟨*U*_CG_⟩ would not give the FG internal energy. Instead, a reformulated expression ⟨*U*_CG_ + *TS*_CG_⟩ could recover the FG internal energy; finding approximations for *S*_CG_ is an active area of research.^51,121,124^

The thermodynamic representability problem becomes more apparent for observable expressions explicitly mapped from the FG to CG ensembles.^49^ For example, if the observable of interest, *A*, only depends upon configurational variables, this inconsistency can be mathematically formulated by examining the difference between *A*_FG_ = ⟨*A*(**r**^*n*^)⟩_**r**^*n*^_ and *A*_CG_ = ⟨*A*_CG_(**R**^*N*^)⟩_**R**^*N*^_. By introducing thermodynamic consistency into *A*_FG_, it can be shown that observables at the CG resolution use equivalent expressions to that of the FG resolution only when the following definition is used:
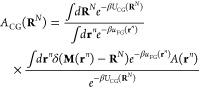
14

However, as can be seen from the complex expression in eq 14, not all observables satisfy this criterion (eq 14), and this often results in *A*_FG_ ≠ *A*_CG_.^49^ There has been considerable interest in determining the observable incompatibility from a rigorous statistical mechanical perspective.^210,211^

Pressure is an example of a thermodynamic observable that does not meet the criterion in the equation above. All FG configurational variables contribute to pressure according to the virial theorem,^157,212^ rather than only the reduced CG configurational variables. As such, the evaluation of pressure in a CG model by simply using the virial expression as if the CG variables were the FG variables has essentially no connection to the pressure of the system at the actual FG level. Stated differently, the “pressure” in the CG model is rather meaningless unless interpreted as a part of a global model in which virtually everything is a model, not just the interactions between the CG particles. On the other hand, if the volume dependence of *U*_CG_ were known, it would be possible to construct a modified virial expression to compute the pressures using only CG variables that are consistent with the FG pressures.^49,146^ One way to resolve this discrepancy is to determine a compatible observable expression using correct basis sets according to the thermodynamic properties of interest. A recent study on determining the correct observable expression for pressure using particle-wise decompositions suggested that such an approach can resolve the thermodynamic representability issue.^213^ In related fashion, Lebold and Noid developed a dual-potential approach that combines both structure- and energy-based variational principles, resulting in a more faithful recapitulation of FG energetics at CG resolution.^195,196^ We believe that this approach is generalizable and that finding appropriate expressions at CG resolution within the parametrized state point forms the basis of CG representability. Moreover, these approaches are expected to further reveal the fluctuations underlying fundamental thermodynamic quantities, e.g., heat capacity and isothermal compressibility, although, similar to CG potentials, they must be able to extrapolate to the system of primary interest.

#### 2-4.D Transferability

In CG modeling, the transferability issue naturally emerges from the differences between the FG and CG Hamiltonians and can be defined as a measure of how predictive or extrapolatory the CG models are to the statistics beyond the parametrized conditions. Since effective bottom-up CG interactions are free energy functions, unlike FG Hamiltonians, the CG interactions will vary at different state points (e.g., pressure, temperature, and composition). They are clearly defined as a function of the thermodynamic state point. For example, the constant *NVT* ensemble is generally chosen in practice. However, this fact does not necessarily mean that bottom-up CG models will have zero transferability. In this regard, imbuing transferability onto bottom-up CG models needs to be carried out in a manner consistent with the underlying thermodynamics. For example, based on the entropy-enthalpy decomposition approach introduced earlier,
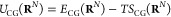
15the changes in entropic contributions to CG interactions *S*_CG_(**R**^*N*^) at different densities or temperatures should be correctly reflected while designing CG models. Most of these conditions are natural variables of free energy (temperature, pressure, volume) and system composition (bulk to mixtures with different ratios). Yet, these conditions are inextricably linked. For example, in mixture conditions, each molecular entity will experience differences in pressure and volume due to the presence of other molecules, resulting in different interaction profiles than that of bulk conditions. We will discuss recent advances in dealing with the transferability issue in Section 3.

### 2-5 Current Challenges in Bottom-up Coarse-Grained Modeling

Currently, major challenges in CG modeling originate from the approximate nature of CG models that aim to faithfully describe the complex many-body correlations and properties of atomistic systems. Here, we present some important challenges faced in these areas that have been and are being actively pursued by a number of researchers in the field.

- *Structural Representability*: How can one design CG models to capture higher-order structural correlations correctly? Most CG models suffer from this issue due to the use of relatively simple pairwise interactions.
- *Thermodynamic Representability*: While structural representability can be directly computed from CG simulations, correct representation of CG thermodynamic properties requires a systematic treatment. For example, how can one obtain comparable pressures, internal energies, or entropies with respect to the FG reference?
- *Dynamical Representability*: Since most bottom-up CG methodologies focus on configurational variables, the resultant CG dynamics is not guaranteed (or even likely) to be consistent with the FG reference. How can one overcome this inconsistency?
- *Transferability*: How can one design bottom-up CG models that can be applied to nonparameterized conditions? This issue is directly related to the applicability of CG models.
- *Machine Learning:* How can one benefit from emerging ML techniques to reduce the complexity underlying CG modeling?

With this in mind, we aim to address each of these issues in the remainder of this Review.

## 3 Toward More Expressive Coarse-Grained Basis Sets

### 3-1 Design Principles for Coarse-Grained Energetics

A bottom-up CG model that follows thermodynamic consistency requires that the effective CG Hamiltonian, *U*_CG_(**R**^*N*^), be exactly equivalent to the many-body PMF in terms of the CG variables.^31−33^ However, it is computationally difficult to derive a many-body expression for *U*_CG_(**R**^*N*^), and many-body potentials are computationally expensive to use. Instead, low-dimensional basis sets are commonly used by adopting commonly used molecular mechanics functional forms, e.g., pairwise nonbonded interactions and bonded interactions (typically up to four-body terms, i.e., dihedrals and impropers).^214−219^ In summary, *U*_CG_(**R**^*N*^) is often approximated as
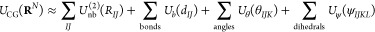
16where *U*_nb_^(2)^(*R*_*IJ*_) is the two-body nonbonded potential that depends upon the distance *R*_*IJ*_ between CG sites *I* and *J*, *U*_*b*_(*d*_*IJ*_) is the two-body bonded potential, *U*_*θ*_(*θ*_*IJK*_) is the three-body angle potential, and *U*_*ψ*_(*ψ*_*IJKL*_) is the four-body dihedral or improper potential.

### 3-2 Current Challenges and Breakthroughs

By approximating the CG force field as eq 16, two types of errors are naturally introduced. The first error is due to the simplified nature of the interaction form (e.g., the pairwise approximation in nonbonded potential). The other error is caused by the inconsistency between FG force fields representing energetics while CG force fields are representing free energies (the CG PMFs). Even though eq 16 asserts that the CG PMF will be only a function of CG configurational variables, *U*_CG_(**R**^*N*^) ignores explicit contributions from other thermodynamic variables (e.g., volume or temperature) as introduced in Section 2-4. It may be typical that the former negatively impacts the structural representability of CG models, whereas the latter limits the thermodynamic representability and transferability of CG models. A summary of these issues in CG force fields is presented in Figure 1.

#### 3-2.A Beyond Pairwise Basis Sets

Due to their pairwise approximate nature, CG models constructed from eq 16 are often unable to reproduce the many-body correlations from the FG reference systems. This problem is exacerbated by the isotropic nature of CG particles (sites) that is commonly adopted upon the CG mapping. By instead introducing virtual sites that are designed to capture such correlations, CG models can be improved while keeping the computational benefit of pairwise basis sets. For example, one can introduce virtual sites to represent complex chemical environments, e.g., the hydration layer surrounding lipid bilayers, where the virtual site interactions can be determined from a hybrid framework that combines structure-based methods and force-based variational principles.^89^ These choices are often more favorable than introducing nonisotropic descriptions for CG particles, e.g., Gay−Berne interactions,^220^ due to the complexity and computational cost of both the CG parametrization and CG simulation. Thus, despite some preliminary efforts in nonisotropic CG particle representation^221−227^ and parametrization strategy,^228^ we will focus on efforts to improve CG models using isotropic CG mapping representations in this section.

Alternatively, based on the many-body expansion,^229^ an improvement can be achieved by introducing higher-order interaction terms in the CG Hamiltonian
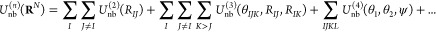
17where *U*_nb_^(*n*)^(·) denotes the *n-*body nonbonded interaction with configurational variables (·). For example, studies of bottom-up 1-site CG water have shown that pairwise interactions are incapable of recapitulating the local structure due to hydrogen bonds but can be recovered with the explicit addition of three-body interactions *U*_nb_^(3)^.^147^ The importance of three-body interactions in molecular systems^230−236^ can be seen by applications of Stillinger−Weber interaction-based models,^237^ including in the top-down CG water model, e.g., mW.^238^

In principle, the aforementioned CG methodologies can be readily applied to determine the interaction parameters for higher-order Hamiltonians, e.g., MS-CG^147,150^ or IMC^239^ for three-body Hamiltonians.^240^ However, introducing many-body interactions inevitably reduces the efficiency gains from CG modeling. In contrast, recent developments have proposed two different generalized approaches to include many-body interactions at a reduced computational cost. The first approach^193,194^ implicitly projects the many-body interaction (up to *N*-body) onto lower-order basis sets based on the conditional probability *p*(***O***_***I***_^(*n*)^ | *R*_*IJ*_) of higher-order configurational variables ***O***_***I***_^(*n*)^
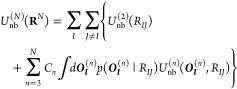
18which can be interpreted as an extended Bogoliubov-Born–Green-Kirkwood-Yvon (BBGKY) hierarchy for the configurational interactions.^241−245^ Recently, eq 18 was applied to water, where the three-body interactions are projected onto effective pairwise interactions, resulting in the Bottom-up Many-body Projected Water (BUMPer) model.^193,194^ The importance of the many-body nature of interactions in water and aqueous systems has been also investigated at the atomistic level, e.g., by the MB-pol potential,^246−248^ or ML approaches.^249^ Notably, BUMPer also highlights the computational efficiency of such approaches while still recapitulating higher-order correlations.

The alternative approach can be realized by explicitly introducing higher-order order parameters (or collective variables) into the CG Hamiltonian. Explicitly evaluating arbitrary higher-order order parameters will often reduce simulation efficiency, but several order parameters that can be computed using pairwise statistics can account for many-body correlations with reduced computational cost. Inspired by many-body Dissipative Particle Dynamics (DPD),^250,251^ there have been active efforts to combine local number density ρ-dependent interactions with conventional pairwise interactions in order to improve the CG Hamiltonian,^252,253^ for example,
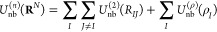
19

Due to notable improvements in modeling implicit solvents^254−256^ and material systems,^257−260^ several papers have reported that utilizing the local density enhances the structural representability of CG models for phase separating or interfacial systems.^261−266^ Taking a step further, one could incorporate variations in the local density in terms of a local density gradient  into eq 19 to accurately describe inhomogeneous systems.^267^

Nevertheless, a direct advantage of eq 19 is that one can introduce any kind of order parameter that can be computed in a pairwise manner, other than local density ρ (e.g., particle orientations,^268,269^ as well as order parameters related to liquid crystals^270^ and glass transitions^271,272^) to accurately describe the corresponding correlations. However, the choice of order parameters used is often determined phenomenologically, and thus, a systematic principle should instead be established. We will provide a systematic framework in Section 4. Finally, more complex, high-dimensional *N*-body order parameters are generally difficult to regress; ML techniques can help solve such nonlinear problems (see Section 6).

#### 3-2.B Transferable Coarse-Grained Force Fields

In the context of CG equations of motion, conjugate forces may additionally contribute to observables (on top of forces due to particle configurations). Since the effective CG interactions obeying thermodynamic consistency are free energy variables,^31−33^ this point of view provides physical insights that introduce conjugate forces in terms of thermodynamic variables.

Early attempts to address temperature transferability were based on heuristics, such as temperature rescaling  for polymers.^273,274^ Recently, more thermodynamically consistent approaches have been pursued using entropy-enthalpy decomposition (eq 15) under the pairwise approximation: *U*_CG_(*R*) = *E*_CG_(*R*) – *TS*_CG_(*R*). By estimating the pairwise thermodynamic functionals (*E*_CG_(*R*), *S*_CG_(*R*)) and extrapolating to nonparameterized temperatures, temperature transferability can be achieved in both constant *NVT* (Helmholtz free energy) and *NPT* (Gibbs free energy) conditions.^51,121,148,275^ Even though eq 15 is a natural extension of the free energy to CG PMFs, the physical meaning of the pairwise thermodynamic functionals *E*_CG_(*R*), *S*_CG_(*R*) remains relatively unclear at present.^276^ A recent report elucidated that these functionals may be deeply connected to the thermodynamic representability issue for entropy.^51,276^ Alternatively, a more direct approach based on statistical mechanics that does not suffer from the ambiguity of pairwise thermodynamic functionals was developed by numerically transferring the phase space expectation value at different temperatures.^145^ It should be noted that reweighting approaches often suffer from inefficient sampling and non-negligible numerical noise.^277,278^ Notably, the dual approach by Lebold and Noid circumvented this limitation in reweighting without sampling other temperatures by employing the least-squares minimization to the energy quantity to obtain *E*_CG_(*R*), which is analogous to force-matching.^195,196^ Recently, Pretti and Shell showed that the CG models constructed from microcanonical basis sets in conjunction with REM for capturing entropy functions can naturally provide temperature transferable CG models by recapitulating atomistic energy fluctuations.^41^ Altogether, current findings and reports, regardless of methodological details, emphasize the role of entropy in temperature transferable CG models.

Temperature transferability is inevitably coupled with pressure transferability in the case of the constant *NPT* ensemble, as both thermodynamic variables affect the system volume.^146,279^ Early improvements were based on rescaling CG interactions with respect to pressure but lacked theoretical rigor. Notably, one can introduce a volume-dependent conjugate force into the CG Hamiltonian, a strategy that dates back to the 1970s for liquid metals.^280^ Das and Andersen showed that introducing volume-dependent interactions *U*_*V*_(*V*) to the CG Hamiltonian can correct the virial pressures in CG models^146^
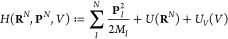
20

This interpretation has been further explored in refs (281−283) to impart transferable CG models while adequately addressing the representability issue. Other than explicitly relying on eq 15 or 20, it is also possible to correctly reflect the changes in CG interactions using order parameters that are coupled to system conditions. One notable example is the Ultra-Coarse-Graining (UCG) approach, which will be discussed in Section 4.

Transferability across composition is a more complicated issue, where both temperature and pressure transferability come into play. The ultimate goal would be to correctly address the reduced pressure in mixture conditions, which is a nonlinear process due to nonideal interactions, and to design the cross-interactions between different molecular moieties. While this direction has not been actively pursued due to its complexity, several preliminary reports have paved the way for developing so-called mixing rules for cross-interactions, indicating that having a correct description of the CG thermodynamic quantities is essential for achieving such transferability.^51,284^ Another free energy-based direction would be to introduce a chemical potential-like term Δμ(*R*, *N*) as an analog to the chemical potential term in the Helmholtz free energy. Eventually, addressing the aforementioned transferability issues will elucidate how to achieve chemical transferability where the CG Hamiltonian can be determined *a priori* by grouping atoms into molecular building blocks (or functional groups) and sampling these groups at various configurations and state variables.^198,285−287^ For example, an extended ensemble approach by Mullinax and Noid suggested the use of topology in composition and chemical transferability.^285^ Combined with ML techniques, such a systematic treatment to predict CG interactions for new molecules is deemed possible by sufficiently sampling numerous small molecules.^287^

#### 3-2.C Holy Grail for a Bottom-up Coarse-Grained Force Field

An accurate CG Hamiltonian should be able to account for both many-body correlations and transferability issues. On the basis of the efforts described above, we argue that the ideal effective CG Hamiltonian may be expressed as
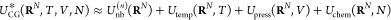
21

In eq 21, *U*_nb_^(*n*)^(**R**^*N*^) accounts for the nonbonded interactions up to *n*-body, which can be explicitly cast based on eq 17 or implicitly described by eq 18. Alternatively, using an appropriate *n*-body order parameter *O*_*I*_ under the pairwise approximation, this term would be  ≈  +  and shares similar mesoscopic physics as the many-body DPD method.^250,251^ The last three terms in eq 21 correspond to temperature-dependent, volume-dependent, and composition-dependent potentials through a conjugate interaction to thermodynamic observables. The first term *U*_nb_^(*n*)^(**R**^*N*^) is independent of state point, while the latter three terms *U*_temp_(**R**^*N*^, *T*), *U*_press_(**R**^*N*^, *V*), and *U*_chem_(**R**^*N*^, *N*) are examples of state-dependent potentials. Both classes are examples of efforts to improve the expressivity of *U*_CG_^*^(**R**^*N*^, *T* , *V*, *N*).

Alternatively, eq 21 can be interpreted as a projected Helmholtz free energy functional along pairwise basis sets. Note that  and Δ*A*_CG_(*V*, *T*, *N*) = ⟨*U*_CG_^*^(**R**^*N*^, *T*, *V*, *N*)⟩. This interpretation is in line with the state-dependent potential derived from the free energy perspective.^288,289^ Recently, some CG models have been developed based on the above principles and shown favorable results, such as the combination of density-dependent interactions with volume-dependent terms^290^ or the UCG models in the mean-field ansatz.^275^

While eq 21 aims to directly determine conjugate force components in free energy expressions, an indirect approach based on the free energy is also possible by applying the perturbations to the conjugate variables of interest in the coarse-graining process.^284^ By deliberately extending the simulation ensemble, matching a response of the system to perturbations has recently shown to sample a broad range of parameters. This wide parameter space with more abundant information allows for determining the most transferable CG interactions by maximizing an information metric, e.g., Fisher information.^291^ In the future, it would be informative to rigorously elucidate the physical nature of these pairwise thermodynamic functionals and their relationship to CG systems at different thermodynamic state points and ensemble conditions.

### 3-3 Mini Outlook

As researchers continue to explore different avenues to increase the expressivity of *U*_CG_, it is important to remain mindful of the trade-offs between model complexity and computational cost, the latter of which includes the cost of parametrization, implementation, and runtime. The development of effective CG interactions bears resemblance to “Jacob’s Ladder” as seen in density functional approximations across quantum chemistry.^292^ Each degree of complexity can be thought of as a new “rung” on the “ladder” that represents the field of CG modeling. We note, however, that climbing successive rungs does not necessarily guarantee improvement in the overall accuracy of a given CG model. For instance, it is possible that the increased complexity enhances the adherence to CG model consistency at the expense of CG thermodynamic representability and transferability.^156^ To avoid these pitfalls, a standardized method to derive, explore, and validate the benefit of increasingly complex basis sets is still needed. Our focus in the next two sections will be to discuss frameworks that can systematically increase the expressivity of CG force fields using theoretical and algorithmic procedures.

## 4 Ultra-Coarse-Graining: Machinery to Generalize Representability and Transferability

### 4-1 Necessity of Generalized Coarse-Grained Framework

In Section 3, we introduced several challenging problems that are intrinsic to the parametrization nature of CG interactions. For different systems, conventional approaches introduced earlier have tackled these problems by altering the CG Hamiltonian form or parametrization strategy case-by-case, which limits general applicability. In order to attain a more generalized approach, the Ultra-Coarse-Grained (UCG) model and methodology were developed from the observation that these problems originate from the inability of conventional CG models to correctly address underlying chemical or physical changes in the reference system.^263,293,294^ The idea underpinning the UCG approaches is to introduce internal quantum-like “states” into the CG sites, and thus, the CG Hamiltonian can effectively account for the driving forces associated with these discrete changes that are missing in conventional CG force field treatments by modulating the internal state interactions (called the “state-wise” interactions). The basic idea of a UCG model is to utilize a kind of isomorphism with quantum mechanics, i.e., in the latter case the system nuclei can evolve on multiple potential energy functions, which in turn depend on the quantum mechanical state space of the system and its underlying dynamics, and this in turn is tied back to the evolution of the dynamics of the nuclei. “Simple” nonadiabatic dynamics with surface hopping between electronic states^295,296^ is one example of this behavior, albeit there can be other examples. The basic notion of the UCG approach is to utilize this quantum isomorphism to increase the expressivity of the CG model so that the influences of the processes that become implicit at the CG level are still included to a certain degree.

The importance of the underlying molecular nature beneath the CG resolution is pronounced in many systems, especially for highly coarsened representations. As indicated by refs (297 and 298) for ATP hydrolysis in actin protein, the missing important molecular details beneath the CG models can affect the free energy landscape and should be incorporated into the pertinent UCG models.^299,300^

### 4-2 Ultra-Coarse-Grained State Dynamics: Practical Realization

**Figure 2:**
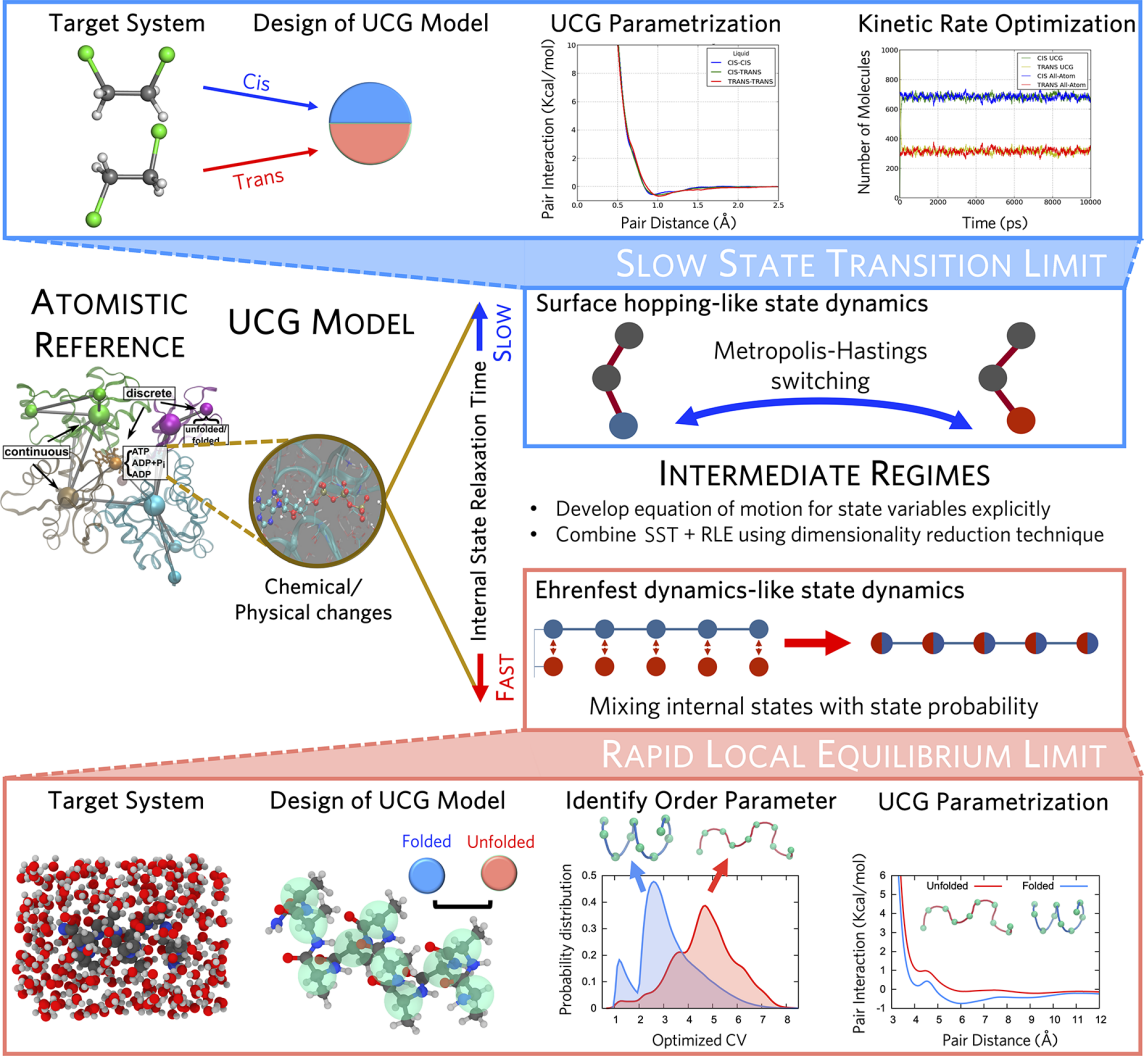
UCG models are designed to capture the chemical or physical changes “beneath” the CG resolution (illustrated upper middle left for ATP hydrolysis in F-actin). Practical design principles of UCG models are based on the relaxation time of internal state dynamics. (1) In the slowest limit (SST), UCG state dynamics can be treated as a kind of surface hopping. An example of this is the gauche- and anti- configurations from 1,2-dichloroethane (top panel). Based on the target system, distinct UCG states are identified, and the UCG models are built by parametrizing the state-wise interactions and optimizing the kinetic rates described by the Metropolis-Hastings algorithm. (2) The internal states at the fastest switching limit (RLE) can be thought to be in quasi-equilibrium, and the Ehrenfest dynamics idea can describe the internal states by mixing them with the state probability. The UCG models are then constructed by identifying the rapidly varying states with the corresponding order parameters. Then, bottom-up CG methodologies can be applied to determine the UCG state-wise interactions. As an example, we depict the solvated peptide here exhibiting folded and unfolded states determined by the optimal CV (bottom panel).

A UCG idea can be mathematically formulated by introducing internal state variables into CG interactions but in principle requires rigorous formulation and parametrization of the correct equation of motion for the state variables, which can significantly increase computational cost.^293^ Since the chemical or physical changes in the molecular system have clearly separated relaxation times, a separation of time scales can be introduced. For example, physical changes such as bond breaking or formation happen much slower than the CG particle mass translations. On the other hand, chemical changes in electronic states or solvation will directly affect the CG system without any relaxation in state dynamics. Thus, instead of seeking a generalized Hamiltonian involving both CG configuration and state variable dynamics, two specific approximations at each time scale limit can be designed,^263,294^ as described below. Figure 2 delineates a design principle for the UCG models in terms of state dynamics.

#### 4-2.A Slow State Transition Limit

Under the slow state transition (SST) limit, the internal states rarely change with the characteristic relaxation time due to conformational transitions that take place at time scales of nanoseconds or longer. A kinetic Metropolis–Hastings-like approach^294^ can thus be utilized to approximate the instantaneous rate of switching from states α to β that are defined by the order parameter *m* (e.g., a dihedral angle ψ in a protein^299,300^).
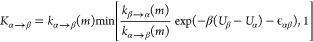
22

In eq 22, the prefactors  and  are determined from the FG simulations, experimental information, or they may be treated phenomenologically; and the model parameter  is introduced to correctly capture the surface hopping-like dynamics from the given state-wise CG interactions  and .

#### 4-2.B Rapid Local Equilibrium Limit

The opposite limit of the SST limit is when internal states undergo rapid state transitions or the so-called rapid local equilibrium (RLE). In this case, in an analogy to Ehrenfest dynamics, the effective UCG Hamiltonian can be expressed as a mixed interaction with respect to its state probabilities that follow quasi-equilibrium distributions. Ideally, one should consider the overall *s*^*N*^ configurations, where *s* is the number of internal states per CG particle, and *N* represents the number of CG particles. However, under the RLE limit, one can choose a local order parameter to decouple state correlations between different particles, resulting in the expression
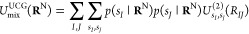
23where *p*(*s*_*I*_|**R**^N^) denotes the probability of CG site *I* being *s*_*I*_, and *U*_*s*_*I*_, *s*_*J*__^(2)^(*R*_*IJ*_) is the state-wise interaction between *s*_*I*_ and *s*_*J*_.^263,301^ Unlike the SST limit, the RLE limit explicitly accounts for the driving forces (the term −∇*p*(*s*_*I*_|**R**^*N*^) in eq 23), and thus, UCG models should be carefully designed using the correct order parameters to extract the nonuniform physical or chemical nature from the system. In this regard, various order parameters suggested from the previous studies can be utilized: external fields, relative particle positions, coordination numbers, and local number densities.^263^ Notably, the UCG force field from eq 23 can be generalized to other CG Hamiltonians, such as the multiconfigurational CG (MCCG)^302^ and conformational surface hopping methods.^303,304^

It must be noted that the UCG approach has already achieved notable success in treating realistic and highly complex biomolecular systems, an example of which is the assembly of the HIV-1 virus capsid from the more than 1,000 copies of its capsid (CA) protein component.^81,84,305^

### 4-3 Structural Representability

#### 4-3.A Chemical Accuracy

Even though most computationally efficient CG models are not able to describe changes in the chemical nature, a key attribute of UCG models in the SST limit, by design, is the ability to explicitly model chemical transformations while avoiding the use of many-body force fields by introducing distinct states that represent different chemical phenomena. This advance has enabled UCG models to capture previously missing conformational transitions between gauche- and anti- conformations in 1,2-dichloroethane^294^ and the characterization of ATP hydrolysis and phosphate-release reactions in actin filaments.^299,300^ In a coarser description (lower resolution), UCG models based largely on the SST limit have been applied to capture the dynamic self-assembly behavior originating from many-protein HIV viral capsids.^81,84,305^ As such, when conformational transitions in complex biomolecules rarely occur, the SST limit can effectively embed the finely detailed chemical nature into a reduced level of representation.

#### 4-3.B Many-Body Correlations

Introducing flexibility into CG interaction forms enables the UCG models to improve the description of the many-body correlations necessary for more accurate CG models. For the SST limit, chemical reactions due to complex many-body correlations that involve many-particle nonbonded and electrostatic interactions can be faithfully folded into the UCG models. Namely, the SST limit implicitly encodes the many-body correlations into distinct state-dependent pairwise UCG potentials, which have a strong similarity to a “polarizable” CG model.^306^

Other types of many-body correlations in terms of nonbonded structures can be practically impossible to address using only pairwise basis sets. We note, as before, that pairwise MS-CG models attempt to capture two-body and three-body correlations concurrently by satisfying the YBG equation,^31^ but three-body correlations and even higher-order correlations are often not well-captured in complex CG systems, e.g., water.^147,165,193,194^ Even though the UCG models at the RLE limit are built upon the pairwise basis sets, these state-wise interactions are linked via local order parameters. By choosing these local order parameters properly, the resultant UCG model can faithfully capture complex many-body correlations. Based on the observation that the local density is an *N-*body property, several studies have reported that the local density-based UCG models can readily reproduce the many-body phenomena of interest. For example, the solvophobic association of hydrophobic solutes due to many-body correlations between solute molecules was captured with the UCG formalism as well as pair correlations and clustering behavior.^263^ The local density can also be an important order parameter for distinguishing different phases in heterogeneous systems. This inhomogeneity is pronounced in interfacial systems, where the conventional CG models failed to properly describe two or more phases at their interfaces. Notably, UCG interface models can differentiate distinct characteristics emergent in the system, e.g., liquid and vapor states in liquid/vapor interfaces with well-reproduced liquid “slab” density profiles and structural correlations.^265^ Extending beyond interfacial UCG models, it was recently demonstrated that the single UCG model is able to encompass the structural correlations emergent from distinct bulk phases, resulting in multiphase CG models.^275^

Since the local density of CG moieties reflects the various chemical and physical natures (e.g., changes in structures and electrostatics that may affect coordination), it is conceivable that the local density can be utilized as a generalized order parameter to indirectly represent the nonuniform nature of a given system. For example, it has been shown that effects of FG hydrogen-bonding interactions can be faithfully captured in a local density-based UCG model by differentiating the donor and acceptor states,^307^ with the hydrogen-bonding not being explicitly resolved at the CG level.

### 4-4 Transferability

The main advantage of UCG modeling not only lies in being able to reproduce important correlations faithfully described by the order parameters but also in its flexible interaction form. This is especially true in the RLE limit, where the UCG Hamiltonian inherits transferability by design; several distinct state-wise interactions are folded into a single Hamiltonian form, allowing for transferring to different state conditions. This flexibility is akin to Ehrenfest dynamics^308^ or the empirical valence bond theory,^309^ where a systematic connection between these different theories has been recently demonstrated.^310^

First, the flexibility of eq 23 enables one to directly employ the MS-CG variational principles to parametrize many state-wise interactions that are distinguished by the imposed order parameter. It has been seen that the parametrized UCG interactions are comparable to bulk MS-CG interactions, confirming the transferability of UCG models. For example, state-wise interactions between denser states in liquid/vapor interfaces are equivalent to bulk liquid interactions, and a similar conclusion holds for liquid/liquid interfaces where the UCG interactions are transferred to liquid mixture systems with different compositions.^265^

Alternatively, one can introduce the already determined MS-CG interactions into the UCG framework and utilize adequate order parameters to distinguish each interaction. For example, one can utilize the local density in order to mix bulk interactions at high temperatures and low temperatures, resulting in temperature transferable UCG models. A recent study showed that such an approach has a direct link to the energy-entropy decomposition under the mean-field ansatz and further demonstrated that one can possibly design phase transferable UCG models by combining liquid and gas phase MS-CG interactions.^275^ Since number density directly responds to the system condition, local density-based UCG models can be good candidates for properly describing the conjugate forces exerted by the thermodynamic variables of the system. For more complex systems exhibiting various conformations, determining the correct order parameter would be the most important step toward transferability; this will potentially benefit from advances in ML techniques that will be described in Section 6.

### 4-5 Mini Outlook

In contrast to conventional CG methodologies, UCG theory provides a generalized framework to greatly enhance the fidelity of CG models, and the practical realization of UCG state dynamics imparts a physical presence of the missing degrees of freedom in terms of internal states at the reduced CG resolution. The UCG method has recently been shown to be successful for describing various systems ranging from liquids to biomolecules, demonstrating its applicability and versatility. We conclude this section by providing open problems for future developments.

- *Intermediate State Dynamics.* Various chemical and physical processes may still exhibit intermediate state dynamics that does not fall into either the SSL or RLE limit. One illustrative example would be proteins that slowly fold but have rapidly changing behaviors due to their interactions with the environment or solvents. Even though one direct direction for modeling intermediate state dynamics would be to incorporate equations of motion for state variables and to develop the corresponding UCG Hamiltonian, we propose a hybrid UCG model as a more efficient alternative by simultaneously accounting for both the SST and RLE limits. For the hybrid UCG model, defining two distinct UCG types that undergo different dynamical limits can benefit from existing dimensionality reduction methods. For example, time-lagged independent component analysis (TICA) can be utilized to extract the most rapidly varying and the slowest varying order parameters.^311^ With the recent success of such dimensionality reduction techniques in Markov State Models (MSM) for CG dynamics modeling,^312−317^ the combination of the UCG methodology with TICA is expected to further extend the range of dynamical transitions explicitly included in CG modeling.
- *Toward Large Biomolecules.* Despite recent developments in UCG models having been mostly focused on relatively simple liquids, the UCG methodology in principle can be extended to larger spatial scales, and MS-CG-based UCG models can be also extended to much larger systems by correctly differentiating the chemical environments using local density parameters. For example, the numerous conformations in protein folding result in complex energy landscapes, e.g., dodecaalanine,^318^ and UCG models that have internal states designed based on the local Cα density could be expected to accurately capture the folded and unfolded states. Similarly, the effect of solvents in a solvated system can be faithfully modulated by solvent density-based UCG models, e.g., lipid bilayers.

On the other hand, for more complex biomolecules, effective large-scale UCG models can be built upon coarser descriptions of state-wise CG interactions. Since the UCG methodology does not specify any of the interaction forms but rather provides a systematic framework for embedding internal state information into CG models, combining several CG interactions from different methodologies with the UCG theory can correctly account for the state dynamics. Recently, the UCG models based on the fluctuation maximization with harmonic interactions were developed for the glutamine-binding protein and lactoferrin and were able to correctly describe the protein conformational transitions.^319^ Relatedly, a network-based UCG model was reported to effectively assess the mechanical properties of microtubules.^320^

## 5 Dynamics of the Coarse-Grained Models

### 5-1 Limitations and Challenges

While the thermodynamic properties of CG models differ from their FG counterparts, thermodynamic representability can be systematically improved based on the FG and CG observable expressions, e.g., pressure and energy. However, dynamical properties pose completely different problems in comparison to those from thermodynamic properties.^52−54^ In this section, we elaborate on such difficulties arising from the dynamics of CG models and showcase recent advances in this area.

In order to assess the relevant dynamical variables in CG representation, the Mori−Zwanzig projection operator formalism can be applied to the microscopic Hamiltonian dynamics at the FG resolution.^321−324^ The idea behind the Mori−Zwanzig formalism is to project the relevant dynamic variables that are left in the CG systems, resulting in a generalized Langevin equation (GLE) form of the equation of motion.^325^ The following integro-differential equation thus faithfully describes the dynamics of the CG model^326−329^
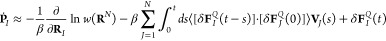
24which is composed of particle conservative force, frictional, and stochastic (random) forces, respectively. The notations in eq 24 are consistent with ref (91), where *w*(**R**^*N*^) is a normalized partition function of the microscopic (renormalized) configurations at **R**^*N*^. See ref (330) for the detailed discussion of the approximation made in eq 24. Therefore, performing the CG simulations under Hamiltonian mechanics using only conservative forces often results in an accelerated CG dynamics due to the missing friction. Note that friction and fluctuations are connected through the second fluctuation dissipation theorem.^91,331^ These fast CG dynamics might be considered advantageous for performing CG simulations, allowing for simulations that span large temporal scales with relatively small time steps. Nevertheless, correct dynamical information from CG simulations is required to evaluate the dynamical properties when making contact with experimental kinetics.

From our perspective, two different approaches may elucidate the correct CG dynamics. The first approach would be to reconstruct the dissipation and fluctuation information from eq 24, such that the velocity correlations, diffusion features, and nonequilibrium properties of the system can be well reproduced. Due to complexity in parametrizing the correct friction and fluctuation terms, an alternative approach can be built upon establishing the correspondence between FG and CG dynamics by analyzing the fast CG dynamics when using Hamiltonian mechanics with the conservative forces along. In this section, we briefly review recent advances in both of these directions. More detailed perspectives for each approach can be found in refs (332) and (333) for molecular CG modeling at equilibrium and in ref (334) for out of equilibrium conditions. Furthermore, ref (335) provides a general review of CG modeling for both in and out of equilibrium conditions.

### 5-2 Incorporating Missing Friction

In order to faithfully represent the frictional and stochastic forces at the reference level, continued attention has been paid to parametrizing the frictional and stochastic forces from the Mori−Zwanzig equation of motion in eq 24.^54,91−93,105,107,327,336−342^ Since the Mori−Zwanzig formalism cannot be directly employed in practical simulations due to its complexity and large computational cost, various approximations have been introduced in the literature to simplify the nature of the CG dynamics, resulting in various types of stochastic differential equations to describe the time propagation of the CG system. The simplest approach one can take is to parametrize the friction coefficient as described by the Langevin equation in which the time correlations and frictional kernels are omitted. For relatively simple CG systems, such an approach has been shown to recapitulate the correct diffusion behavior from the FG level.^54^ Nevertheless, the Langevin equation is a rough approximation of the complete dynamical behavior of CG models.

A more accurate description of frictional and stochastic forces present in the CG equation of motion can be established in two steps. First, one needs to choose an appropriate stochastic differential equation as the equation of motion. Then, based on the chosen equation of motion, one needs to parametrize the friction kernels and associated stochastic forces in a “bottom-up” manner. Typically, the Mori−Zwanzig equation of motion is approximated using a single-particle GLE or DPD-like equation of motion. A single-particle GLE assumes that there are no spatial correlations for the random forces acting on different CG particles. Such an approximation reduces eq 24 into a more tractable form that allows for developing various parametrization methods,^105,341,343−345^ but missing pairwise nature in such approximation violates macroscopic physical principles by not conserving the momentum. Conservation of momentum is particularly important for reproducing the long-time tail of velocity autocorrelations resulting from the hydrodynamic effect.^346^ This discrepancy can be correctly addressed by introducing a fluid mechanical description. For example, the smoothed particle hydrodynamics (SPH)^347−349^ and the smoothed DPD^350,351^ based on the discretized Navier–Stokes equation can resolve the momentum conservation issue by introducing pairwise frictional kernels to eq 24, yet most fluid mechanics-based approaches often suffer from the top-down nature. Thus, one should carefully choose the appropriate physical descriptions at the desired resolution to embed into the CG model in order to reduce eq 24 into an approximate stochastic differential equation.

Once the form of the CG equation of motion is chosen, the remaining step is to determine the friction kernels to be consistent with the FG reference. Here, we provide a brief discussion on bottom-up approaches for parametrizing these nonconservative interactions. For bonded systems, e.g., star polymers, a pairwise decomposition of instantaneous forces into parallel and perpendicular directions at the FG level is possible at the CG resolution. Practically, Hijon and Español introduced the so-called “constraint dynamics” technique to extract the pair decomposed forces under the Markovian DPD equation of motion.^92^ This was further extended to the non-Markovian DPD regime by Yoshimoto,^338^ and in recent years, Karniadakis and co-workers have established a systematic parametrization of GLE and DPD equations of motion for both Markovian and non-Markovian limits.^93,337,352−354^ In this case, the friction kernels are readily obtained from the stochastic forces, allowing direct utilization of the Mori−Zwanzig formalism. However, pairwise decomposition of instantaneous forces is only feasible for bonded systems, and thus, this approach cannot be applied to unbonded systems.

Alternatively, the friction kernel can be constructed indirectly by inverting the time correlation functions from the FG reference.^93,337,338,353^ In practice, this inverse approach matches the pairwise velocity autocorrelation function and force–velocity cross-correlation function, resulting in the Volterra integral equation.^344^ Still, most of these approaches are limited to bonded systems. Notably, a recent breakthrough for constructing friction kernels of unbonded fluids was reported using the dynamic mapping approach.^107^ By estimating the instantaneous forces on dynamic blobs based on the velocity Verlet algorithm,^355^ conservative forces in the form of the many-body DPD interactions^250,251^ were determined using the MS-CG principle. The deconvolution of correlation functions was then applied to derive a DPD-like equation of motion in Markovian and non-Markovian limits. This approach further establishes the bottom-up link between the microscopic origins of fluids and macroscopic physics. Such a bottom-up inference of frictional and stochastic interactions has not been widely investigated due to its complexity but remains a promising direction for future research. For example, using the REM framework, Español and Zuñiga designed a variational approach to infer drift and diffusion terms in the Fokker–Planck equation.^356^ Another interesting extension of the MS-CG method was developed by Davtyan, Andersen, and Voth, where they introduced fictitious particles and coupled them with CG sites to effectively introduce a memory kernel under the GLE,^339,340^ which shares a similar physical idea with the auxiliary model later developed by Karniadakis and co-workers.^353^

### 5-3 Understanding Accelerated Hamiltonian Dynamics

Alternatively, a computationally less expensive yet challenging direction would be to perform CG simulations under Hamiltonian mechanics and then elucidate how the accelerated CG diffusion is related to the FG (reference) dynamics through various rescaling approaches.

#### 5-3.A Time Rescaling

A naïve yet straightforward approach is to think of the CG time scales as uniformly accelerated time with respect to the physical time of the FG reference. This uniform time rescaling approach assumes that the frictional forces are not dependent on time, and thus, the Mori−Zwanzig projection operator can remove the configuration, momentum, and time dependence in the friction kernel.^327^ This approach has been reported for polymer systems at different resolutions and chain lengths but with limited applicability due to the strong assumptions made.^357−364^ We note that even though one could assume a uniform scaling ratio and estimate such value naïvely based on the FG and CG diffusion, there is no theoretical guarantee that such a factor exists^365^ and may necessitate an explicit consideration of the Mori−Zwanzig formalism.^366−369^ Also, the uniform scalar friction term obtained from the scaling factor itself is a many-body quantity (renormalized memory kernel) and differs by system conditions, e.g., thermodynamic state point, and hinders its applicability to other chemical systems.

#### 5-3.B Free Energy Landscape

Inspired by the energy-landscape theory,^370,371^ e.g., protein folding,^372^ the free energy landscape approach aims to address the dynamical properties underlying barrier-crossing dynamics by correctly representing the CG energy landscape. While the barrier-crossing dynamics is quite different from the microscopic dynamics of the system, recent advances have elucidated the structural-kinetic-thermodynamic relationships for helix–coil transitions of helix-forming peptides.^373^ More importantly, such approaches have been shown to have a direct link to the MSMs, where the system dynamics is represented by transitions between microstates.^374−377^ Notably, based on variational approaches to understanding the conformational dynamics and then follow-up work,^378,379^ Nüske et al. have developed a spectral matching method that targets the dynamical propagator of CG systems, resulting in correct long-time dynamics.^380^ Outside of the MSM-based framework, Rudzsinki and Bereau have developed the Bayesian dynamical reweighting scheme^381^ to correctly recapitulate the kinetics of CG peptides.^382^ These pioneering efforts in barrier-cross dynamics have highlighted how existing observed deficiencies in CG dynamics and kinetics may be due to not only incorrect CG equations of motion but also inaccuracies in the approximation of the conservative forces (i.e., the many-body CG variable PMF).

#### 5-3.C Excess Entropy Scaling

Another important recent advance in CG dynamics has been an attempt to understand accelerated CG dynamics using the excess entropy scaling relationship. First proposed by Rosenfeld,^383−385^ the excess entropy scaling relationship is an empirical, semiquantitative relationship that links the dynamic property of the system D* to its molar excess entropy *s*_*ex*_, which is the entropy difference between the system and ideal gas, such that
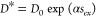
25

To date, only a handful of studies paid attention to the potential usefulness of eq 25 to assess CG dynamics in terms of entropy, e.g., the perspective from the REM,^39^ and until recently, employing eq 25 for CG systems had not been extensively pursued. This is mainly because Rosenfeld scaling is not established from first-principle physics, limiting the applicability of this scaling relationship.^383,386−391^ The empirical nature of this scaling relationship further exacerbates this limitation when applied to CG dynamics. First, it is not guaranteed that the FG and CG systems will obey the same scaling relationship, i.e., α_FG_ α_CG_. Also, the correspondence between *D*_FG_^*^ and *D*_CG_^*^ is still unclear because there is no physical explanation for the *D*_0_ term, which is the “entropy-free” coefficient from eq 25.

**Figure 3:**
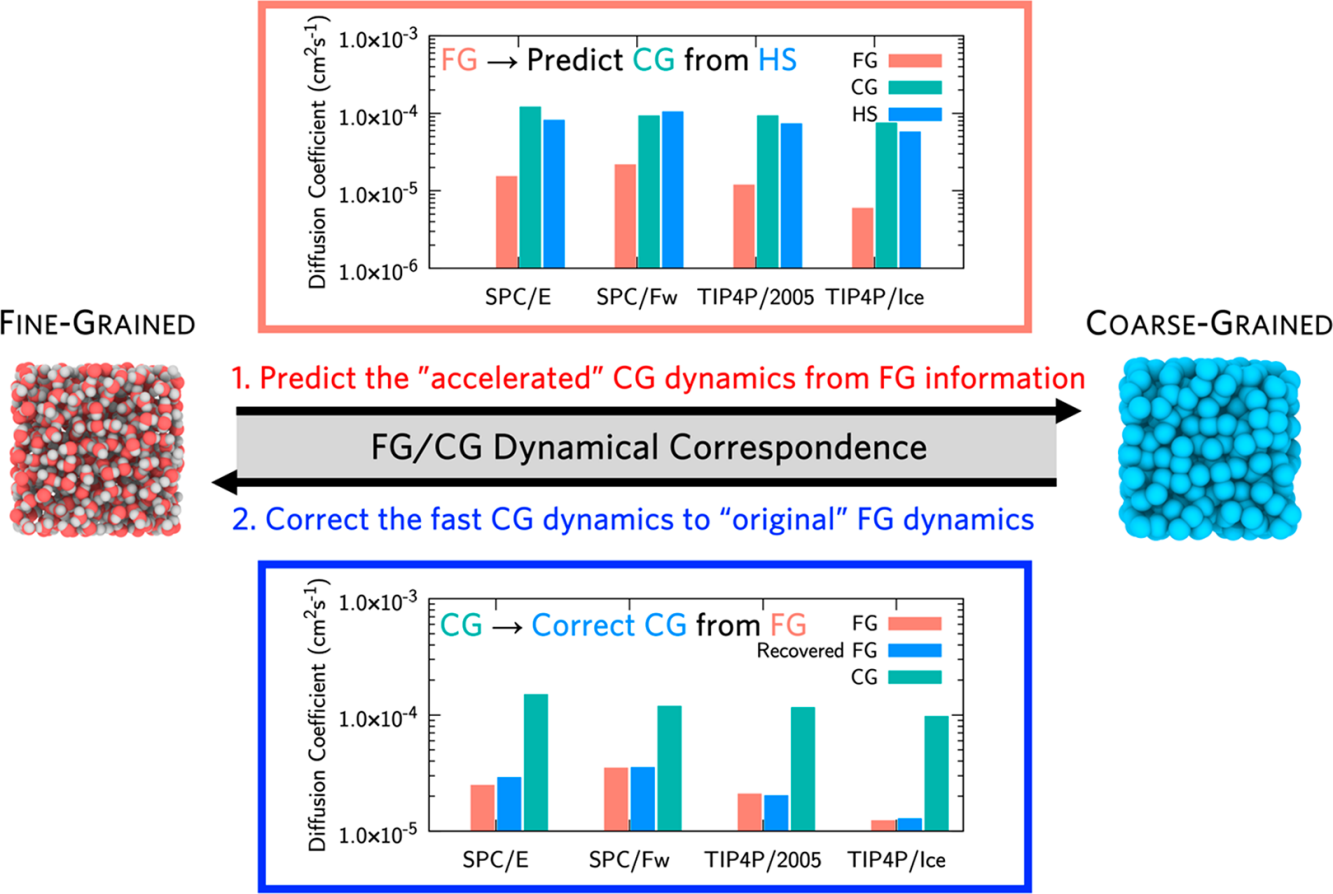
Summary of the excess entropy-based approach to achieve dynamical representability under Hamiltonian mechanics. The dynamical representability of CG models can be addressed by having a dynamical correspondence between the CG and FG models. In this case, without correct fluctuations, CG dynamics is spuriously accelerated compared to the reference FG dynamics (e.g., diffusion coefficients in this figure). Therefore, the ultimate goal in dynamical correspondence would be to address both directions across the FG and CG systems: (1) Predict the accelerated CG dynamics from the FG information and (2) correct the fast CG dynamics to match the original FG dynamics by observing the missing degrees of freedom upon the coarse-graining process. We depict the molecular liquids (water in this case) at the single-site CG resolution as an example, where the hard sphere (HS) mapping theory can achieve (1) and incorporating the missing rotational information can address (2) (recovered FG).

Recent progress on the excess entropy scaling has addressed these aforementioned problems,^392−394^ starting from the excess entropy difference between the FG and CG systems, known as the mapping entropy.^51^ By computing the excess entropy based on earlier arguments from Karplus, Lazaridis, and Zielkiewicz,^395,396^ ref (392) has confirmed that the universal scaling relationship will hold for the same molecular systems upon the coarse-graining process for fluids. In addition to unraveling the universality of the Rosenfeld scaling in CG models, it was recently demonstrated that *D*_0_ at the single CG-site resolution can be physically understood from the hard sphere nature of CG models of liquids, resulting in the analytical form of *D*_0_ determined by specific equations of state.^393^ Such an approach requires an additional layer of coarsening of CG systems to describe them as dynamically consistent hard spheres, and it has been demonstrated that classical perturbation theory can determine the effective hard sphere by mapping the short-range repulsions (e.g., Barker−Henderson theory^397,398^) or long-wavelength fluctuations (e.g., Weeks–Chandler–Andersen theory^399−401^ or fluctuation matching^393^). While the hard sphere treatment of CG systems determines the *D*_0_^CG^, the entropy-free diffusion coefficient for FG systems is usually larger than *D*_0_^CG^. This discrepancy can be understood from the degrees of freedom that are missing at the CG resolution.^394^ By incorporating the missing rotations and vibrations back into the single CG-site resolution, the complete dynamic correspondence between the FG and CG systems can be recapitulated for liquids. In practice, then, one may (1) predict the accelerated CG diffusion under the Hamiltonian mechanics by estimating the *D*_0_^CG^ or (2) recover the reference FG diffusion from the CG level by incorporating the missing diffusion into the translational CG diffusion. However, these recent findings have primarily focused on relatively simple CG systems of liquids, so considerable effort to extend such dynamic correspondence to nontrivial, complex CG systems, e.g., biomolecules, will be a challenge and should be pursued. A detailed description of the dynamical correspondence between the FG and CG systems using the excess entropy scaling is given in Figure 3.

### 5-4 Mini Outlook and Future Challenges

In order to correctly address dynamical representability in bottom-up CG models, recent studies have examined the dynamical properties of CG models using both GLE-like equations of motion and rescaled Hamiltonian mechanics. While the GLE description provides a rigorous statistical mechanical description of CG dynamics, complexity and numerical stability are currently a bottleneck for application to complex molecular CG systems. In addition, similar to the conservative interactions, dissipative and stochastic forces present problems with transferability. The majority of attempts has only been tested on uniform single-component fluids, as multicomponent systems tend to face transferability issues. Also, in the same context as the transferability of the conservative forces (i.e., the many-body CG PMF), thermodynamic transferability of dynamic properties should be addressed to impart a high fidelity CG model. This particular area has not been extensively explored at the current stage, yet several preliminary directions have provided potential directions: dynamical rescaling,^366,367^ energy renormalization,^402−406^ and transfer learning using ML techniques.^407^

On the other hand, understanding accelerated CG dynamics produced using Hamiltonian mechanics can lessen complications from frictional interactions by introducing *ad hoc* physical scaling principles, e.g., excess entropy scaling. In this regard, rigorous physical scaling principles beyond the hard sphere description for explaining the excess entropy scaling are a promising area for future research. A few possible directions are based on the mode coupling theory^408,409^ and transition state theory.^410^ A grand goal in CG dynamics would be to correctly understand and faithfully reproduce transport phenomena under nonequilibrium conditions. Continuous development of a rigorous and practical bottom-up CG theory based on nonequilibrium statistical mechanics would therefore pave the way to a new era of CG modeling.

## 6 Machine Learning and Molecular Coarse-Graining

### 6-1 When Bottom-up CG Modeling Meets Machine Learning

ML is a subfield of artificial intelligence that uses algorithms to study and analyze data.^411^ For example, in order to design an automated method that can determine the content of a picture by inspecting its pixels, e.g., whether the image contains a cat or a dog, two strategies could be considered. First, an algorithm could be explicitly programmed to analyze each pixel in the image to determine whether the picture is more similar to a cat or a dog—an admittedly difficult task. On the other hand, one could use ML procedures to extract patterns from a large set of images that are already labeled as containing a cat or dog. This ML-based approach would then use this labeled data to learn the connection between individual pixels and the overall content of the image, resulting in a new algorithm that is able to discern overall picture content. In other words, ML approaches can leverage high volumes of data to perform tasks that seem intractable when using other strategies. These techniques, however, naturally require a large amount of data to succeed and produce solutions whose quality and accuracy fundamentally depend on the quality of the data used for parametrization. Furthermore, the produced solutions may be opaque and extrapolate poorly to cases outside the data used for parametrization, creating a natural barrier to transferability. Nevertheless, the ability to create complex algorithms from data has revolutionized a number of areas such as computer vision and advertisement targeting.^412^

Since bottom-up CG approaches focus on learning patterns from data sets generated from FG simulations, it should come as no surprise that algorithms developed in ML have found use in molecular CG modeling. Given the similarities between CG and FG models, a natural extension is to employ atomistic ML techniques for CG systems. Our particular interest in this subsection, however, is to describe applications of ML to CG modeling with a focus on how these methods differ from ML used in the atomistic setting. For a thorough survey of atomistic (as well as some CG) ML methods, we refer readers to recent reviews.^60,413−418^

### 6-2 Machine Learned CG Force fields

#### 6-2.A Machine Learning Design Principles

In the language typical to ML, the MD-based CG and atomistic models discussed in this article fall under a class of methods referred to as energy-based models (EBMs).^419,420^ These EBMs are algorithms that describe a distribution by specifying its probability up to an unknown normalizing constant (CG and atomistic force fields are naturally related to the “energy” term of EBMs). However, the data available for training and the need for scientific extrapolation create a specialized domain with its own approaches and difficulties: similar to the atomistic setting, the availability of a noisy estimate of the forces of the many-body PMFs provides an important avenue for parametrization not available in most EBM applications, and the need for physical transferability impedes the use of many EBM architectures. These two key details underpin the training and design of force fields. Despite these differences, various function representation approaches have been successfully adapted from ML to increase the flexibility and accuracy of atomistic force fields for over two decades,^411^ and many of these same methods have now been transferred to the CG resolution. Recent reviews have detailed several ML-based force field approaches, including several kernel-based methods such as the Gaussian Approximation Potential by John and Csányi,^421^ the method introduced by Scherer et al. that uses Gaussian process regression projected onto tabulated potentials,^422^ and Gradient Domain ML-based methods as described by Chmiela et al.^423^ and Wang et al.^424^

A growing number of CG neural network approaches have also been introduced for estimating the many-body CG variable PMFs, likely beginning with Lemke and Peter who developed a convolutional neural network-based approach to learn corrections to an existing CG force field through ideas connected with noise contrastive estimation and adversarial learning.^57,425,426^ We also note that publications such as Schneider et al.^427^ similarly proposed using neural networks to capture free energy surfaces but did not do so for a high-dimensional particle representation. The Deep CG Potential (DeePCG), introduced by Zhang et al.,^428^ was the first to adapt more traditional ideas from the atomistic force field community,^429^ followed by CGnet.^59^ We note that while typically used with highly general feature sets, these same approaches could also be applied to custom high-dimensional order parameters as discussed in Section 3-2. Wang and Bombarelli subsequently introduced an autoencoder augmented approach.^424^ Traditional autoencoders are commonly used in data compression as they temporarily reduce the feature space within the network.^430−432^ This bottleneck feature allows the neural network to find a CG mapping operator before force-matching.^433^ In addition, CGSchNet developed by Husic et al.^434^ and the recent work by Ruza et al.^435^ have introduced graph neural network-based methods to CG force field development. These approaches can be viewed as combining multilayer perceptron-based approaches (e.g., CGnet), which produce a CG force field using user-supplied featurization, with SchNet,^436^ a graph neural network architecture originally designed to reproduce *ab initio* forces and energies, and a compatible simulation engine, TorchMD.^437^ This use of graph neural networks requires no molecular featurization to be supplied by the user, as the network learns its own features via its graph subnetwork. This also has the added benefit of ideally making CGSchNet transferable across system composition and accurately modeling the solvation environment for biomolecules in a novel manner with ISSNet.^438^ We note that graph neural network architectures^439^ and graph-based approaches^440^ have additionally found widespread use in other molecular tasks such as the selection of CG mapping operators and automatic sampling of atomistic configurations.^441,442^ The CGnet architecture has also been adapted to only consider many-body interactions up to a specified order. For example, it is shown that even 5-body interactions notably improve the quality of the resulting CG model when studying a small protein.^443^

#### 6-2.B Parametrization

When parametrizing an atomistic model to match *ab initio* approaches, the force field is often trained via regression to reproduce the connection between molecular configurations and the energies and forces present in the reference data set. While the initial data set may often be generated via MD using a reference energy function, this is not required; in fact, configurations outside the stable basins of the reference system are often critical for reproducing the barriers fundamental to chemical behavior.^416,417,444−448^ These additional structures are either included through human intervention or actively added to the reference set through a variety of strategies, one of which is of particular importance to the current discussion when considering neural networks: query by committee (QBC).^417^ In QBC, multiple neural networks are trained with varying initial optimization conditions on a given reference data set.^449^ While each of these neural networks can typically reproduce energies on the reference data set, their predictions outside this domain may differ. This disagreement is then used as an engine for adding new structures to the reference data set: If a candidate structure results in disagreement among the various neural networks in the committee, a reference *ab initio* calculation is performed, and the configuration is used for parametrization. While QBC is an important technique, its construction emphasizes how neural networks trained on finite samples (especially those drawn from the reference Boltzmann distribution, as is often used for the initial *ab initio* data set) often exhibit inaccuracy outside the stable basins of the reference system—thereby highlighting the importance of complex sampling for high-dimensional force field parametrization.

While this ability to add particular configurations (along with energies and forces) to a data set has proven to be critical to the creation of high-dimensional atomistic force fields, this route has not typically been pursued in the creation of CG force fields. In order to understand the barriers to doing so, it is helpful to compare the settings in which CG force fields are parametrized in comparison to their atomistic counterparts. The atomistic configurational energy corresponds to the many-body PMF in terms of the CG variables, which is extremely difficult to evaluate in practice. However, for the force-based parametrization strategy given in Section 2-3, it is often straightforward to create a noisy version of the forces given by the many-body PMF using the forces in an atomistic trajectory. This noisy signal is compatible with techniques such as least-squares regression, partially reproducing the setting typical to atomistic modeling. However, it is unclear how to add a single CG configuration to a force-based reference data set unless conditional sampling is used, as forces from a single configuration have no clear connection to those of the many-body PMF—only the conditional mean over all such configurations weighted according to the Boltzmann distribution does. The modeler is thus often forced to either select the corresponding atomistic configuration from said conditional distribution by running constrained atomistic MD or to use such constrained MD to directly provide noiseless forces at arbitrary CG configurations, e.g., using blue moon sampling.^421,450^ Doing so creates an avenue to use active sampling strategies such as those described in the previous paragraph.

However, often due to computational reasons, most studies instead resort to canonically distributed nonconstrained atomistic reference samples for parametrization (the use of non-Boltzmann distributed data sets to parametrize CG models has been performed recently^421,452^ but does not seem to be commonplace). This lack of non-Boltzmann sampling, when combined with the similarity between the model architectures used for atomistic and CG force fields and the nature of active sampling strategies, seems to imply that current highly flexible CG potentials trained using forces may persistently face difficulties outside the stable basins of the system under study. The situation for CG models may indeed be sometimes worse than the atomistic case as implied by the previously mentioned results of Wang et al.,^443^ where high order CG potential terms were required to reproduce atomistic results, implying that low capacity model representations may often be insufficient. Despite this, however, the preliminary success of the results discussed in earlier subsections provides hope for quantitatively accurate CG force fields using ML-based algorithms.

Additional strategies beyond force-matching have been developed (see Section 2) to parametrize CG models. These approaches generally aim to either reproduce a particular correlation (low-dimensional marginal distribution) or a high-dimensional distribution described by a reference atomistic trajectory mapped to the CG resolution.^168,169,453,454^ While not the focus of this Review, we note that the direct inversion of radial distribution functions to pair potentials using neural networks has shown to be of repeated interest and represents a route to parametrization requiring no additional reference data once successful CG methods are established for similar systems. Since the forces present in atomistic trajectories are not referenced in these approaches, they are sometimes applicable to general EBMs when the model architectures are compatible. For example, the optimization procedure underpinning REM in the CG literature closely matches the maximum likelihood training of EBMs,^420,455^ although, in the CG case, the supporting theory^35^ has a stronger multiresolution focus. The use of classification to differentiate between data produced by a candidate CG force field and that of the atomistic reference has led to both additive updates reminiscent of noise contrastive estimation^57,425^ as well as the adversarial strategies imitating those found in Generative Adversarial Networks (GANs).^58,426^ Despite these initial connections, however, explicit cross-pollination between these fields remains sparse and is an area for future development.

### 6-3 Machine Learning-based Analysis of CG Models

#### 6-3.A Recent Advances in Machine Learning

CG simulations, similar to their atomistic counterparts, create large amounts of data, and transforming this data into knowledge and understanding is of utmost importance and yet a difficult task. As mentioned in Section 6-1, a large number of ML-based techniques have been developed to understand the data produced by atomistic simulations and are generally also applicable at the CG resolution.^60,413−417^ We refer interested readers to the aforementioned reviews. In this section, we instead focus on two novel applications of ML that focus on problems common to CG simulation: the lack of atomistic detail and the inability to identify high-dimensional structural representability issues.

#### 6-3.B Sample Generation and Reconstruction

CG models are more efficient than atomistic simulations due to their reduced resolution, yet as posed earlier, this simplification inhibits understanding the molecular driving forces underpinning emergent behavior. Approaches that reintroduce atomistic details into CG configurations (aptly referred to as “backmapping” methods) provide strategies to take advantage of the efficiency of CG models while maintaining a clear atomistic picture. Several ML-based techniques have recently been developed to backmap CG data. Most of these approaches use generative models^456^ or GANs in order to create samples at the atomistic resolution that are consistent with a given CG sample. GANs work by simultaneously training two neural networks which “compete” against each other.^426^ One network (the “generator”) is trained in the task of creating the backmapped configurations, while the other (the “adversary”) is trained to classify structures as being generated either via atomistic molecular dynamics or the generator network. Over the course of training, the generator ideally improves to the point that a fully trained adversary can no longer distinguish between the two sources of samples. In this ideal case, the generator produces atomistic samples that are Boltzmann distributed conditioned on a given CG sample. In application, similar to ML-based CG force fields, the network could first be trained on a smaller system and then applied in a larger context, providing a way to atomistically interpret emergent behavior discovered in CG simulations.

There are currently multiple examples of GAN-based backmapping methods. One method introduced by Li et al. uses the Pix2Pix network architecture which was originally designed as a style transfer network for images.^457^ By converting CG positions into a two-dimensional image, the method is able to perform backmapping with minimal additions to an already existing framework.^458^ Another method by Stieffenhoffer, Wand, and Bereau utilizes a new network architecture designed specifically for the task of backmapping.^459^ In this so-called deepBackmap approach, the network not only sees CG configurations but also has access to force field information, allowing higher energy structures to be directly penalized. The network also distinguishes between different chemical groups such as aromatic rings and backbone atoms and additionally builds the high-resolution structure one particle at a time, which allows information from previous atoms to inform the placement of later atoms. Recently, this method showed promising results for complicated systems such as polymer melts and can translate well to crystalline structures.^286^ We note that such stochastic backmapping methods would also allow the force-based active learning strategies previously described to be performed at a minimal cost.

We also note that a number of ML methods other than GANs can be also employed to perform backmapping. To note, various backmapping methods have been developed by utilizing traditional supervised/unsupervised methods^460^ (e.g., graph methods and PCA) or advanced ML methodologies, including Bayesian inference,^461^ Gaussian process regression,^462^ and autoencoders.^433^ In particular, the approach of directly generating molecular configurations without extensive MD has similarly been exploited by Boltzmann Generators, which are trained on small amounts of molecular dynamics data and the atomistic force field in an unconditional manner.^463^ This technique, while not currently implemented for CG data, could similarly be adapted to the backmapping domain and would have the advantage of taking into account atomistic force field information in a rigorous manner.

#### 6-3.C Ensemble Comparison

Even though bottom-up CG models are rigorously built upon FG statistics, many CG models have structural representability issues. In addition, the high-dimensional parametrizations typical to bottom-up models can make it difficult to intuit how two approximate CG models differ beyond their qualitative or projected behavior. When parametrizing atomistic force fields, the per-configuration error in the energy or forces is known for each entry in the reference data set, and these known discrepancies can provide intuition on the regions of phase space well-described by the model at hand. Unfortunately, this type of analysis is not possible for bottom-up CG models, as neither the true many-body CG variable PMF nor its gradients are typically known (although, as noted previously, the gradients may be estimated by constrained simulation techniques). Instead, the validation of CG models is generally limited to comparing their performance using low-dimensional free energy surfaces (i.e., marginal distributions), leaving the differences in their full high-dimensional behavior relatively undescribed.

In this regard, recent work^464^ has proposed a different strategy for capturing and describing the errors present in parametrized CG models. The output of a calibrated classifier trained to differentiate between configurations in the reference atomistic data set and those produced by a candidate CG model can be transformed into an estimate of the difference in the CG force field and the true many-body PMF at each configuration (and, if the classifier is differentiable, the difference in the forces at these configurations), variationally recapturing the information present when dealing with atomistic force fields. Furthermore, the applicability of classifiers in this domain opens the door to exploiting techniques from explainable ML in order to interpret the inferred configurational errors. These same ideas are also applicable to resolutions coarser than that of the CG force field and provide an avenue for understanding classification-based training techniques, such as those in Lemke and Peter’s work as well as in adversarial-residual-CG (ARCG).^57,58^

**Figure 4:**
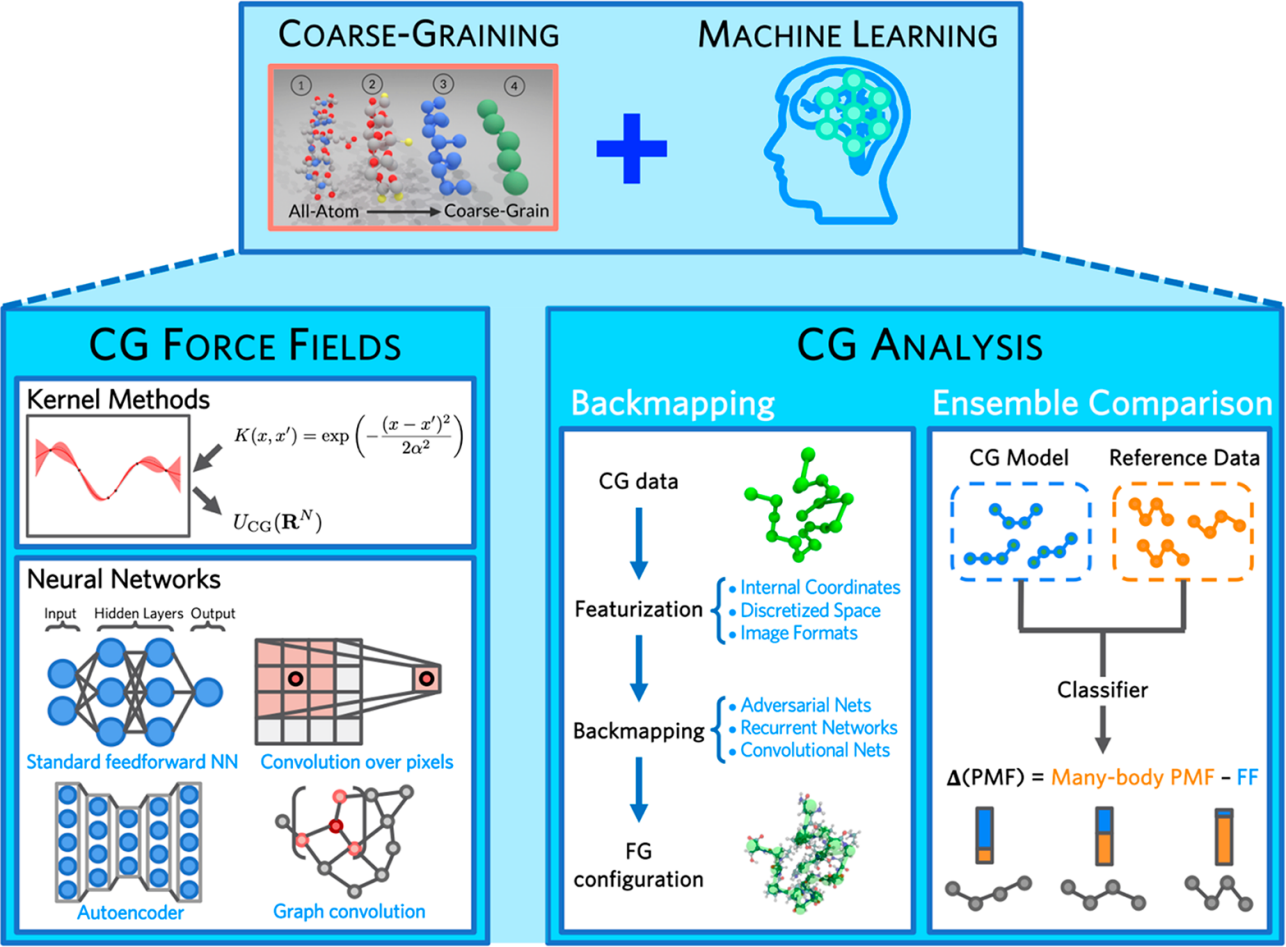
A summary of machine learning methods related to CG modeling. Kernel methods utilize a covariance function to specify a random process which can be used as a nonlinear estimator for CG potentials (left panel, top). Artificial neural networks (ANNs) can also be used to generate nonlinear force fields. A variety of ANN architectures have been developed with applications in CG modeling, such as autoencoders which compress the data stream before expanding it, which has natural connections to CG methods, and graph convolutions, which apply filters over graphs such as molecular topologies (left panel, bottom). Machine learning has also found uses in analyzing CG models and trajectories. Neural networks can be used to backmap CG configurations (right panel, left). Classifiers, especially interpretable ones, can be used to differentiate between configurations generated by different models (such as a CG model and the reference data it was parametrized from) and also provide specific information about how the ensembles are different (right panel, right).

We summarize the current advances in ML-based CG approaches in Figure 4.

### 6-4 Mini Outlook

Both bottom-up CG and ML use algorithms to discern patterns in data. This similarity has motivated the application of a variety of ML algorithms in CG frameworks, ranging from novel force field approaches to systematic methods for reintroducing atomistic detail. Collectively, this increased expressivity can produce atomistic explanations for previously uninvestigated phenomena. However, this increase in accuracy has come with a loss in model transparency (whether this trade-off is inevitable is a topic of debate).^465,466^ In situations where extrapolation and physical intuition are critical, this trade-off can create problems. For example, while bottom-up ML CG models may recapitulate the ensemble it is parametrized to match, are the derived force field parameters transferable to the primary system of interest where emergent behavior is expected? Do backmapping approaches parametrized in the stable basins of a system extrapolate correctly to the transition regions? If a model is inaccurate in portions of phase space, can this be predicted and scientifically understood? As the parametrization of bottom-up methods is often based on reference data sets that inherently do not guarantee the performance of CG simulations, these questions are critical to the many ML-based approaches currently in development. These issues, of course, are not new to the bottom-up CG community (see Section 2) or the ML community.^467−472^ Unfortunately, the nature of molecular models with high-dimensional parametrizations producing molecular configurations in a high-dimensional phase space makes these issues even more pertinent. Nevertheless, the initial successes mentioned in previous paragraphs, along with the successful creation of increasingly general purpose atomistic potentials,^473^ motivate future development for all the applications described in this section. Once the transferability of an approach has been systematically established (ideally through a combination of transparency and application), further work can focus on computational efficiency and ease of use, ideally leading to a class of CG models that underpin a new wave of research devoted to previously insurmountable scientific problems. In general, one may also expect an explosion of new literature in the future in terms of ML methods applied to coarse-graining.

## 7 Concluding Remarks and Perspective

For the past two decades, bottom-up CG models have been developed by studying the microscopic origins underlying many macroscopic processes and have emerged as efficient, powerful, and multiscale computational tools in several fields of science. However, due to the enormous complexity of atomistic systems, statistical mechanics-driven CG modeling has primarily only been possible for relatively simple systems, e.g., liquids and small peptides, using various practical approximations from *ad hoc* design principles. To move toward true “multiscale” models, such bottom-up principles should maintain accuracy across different physical scales. Notably, some recent advances are pushing this limit forward to much smaller (quantum regime) and larger (meso- to macro-scopic) regimes. An extension of the MS-CG framework to the quantum regime,^474^ described by quantum Boltzmann statistics, corroborates that such a multiscale treatment is possible in one extreme, while the other extreme toward macroscale CG modeling has been actively pursued via mesoscopic fluids.^105,107^ Similarly, the Electronic Coarse-Graining (ECG) method^475,476^ has been developed to target configurationally dependent electronic structure and applied to semiconductors^477^ and optoelectronic materials.^478^ These advances promise to significantly extend the spatiotemporal scale of systematically parametrized simulations, which currently encompass biological entities comprising millions to billions of atoms, e.g., the SARS-CoV-2 virion.^479^ When bridging across distinctly different scales, transferability between these scales is critical to reconcile different emergent physics within a single unified model. While at an early stage, recent success in quantum mechanics/CG molecular mechanics (QM/CG-MM),^480,481^ which encompasses both quantum and molecular regimes, implies that it is possible to design CG mappings, equations of motion, and energetics based on the scale of interest. In the other limit, a promising direction would be to incorporate classical field theory into the bottom-up CG framework.^482^

While future efforts should focus on extending the multiscale regime, an equal amount of attention might be spent developing new CG theories at the molecular level. Possible directions for new CG methodologies include the following: (1) stable and extensible nonlinear CG mappings for complex mesoscale systems, (2) fully expressive CG energetics, (3) a more complete description of UCG state dynamics, (4) a more complete dynamic representability between the FG and CG systems, and (5) advanced ML-based CG methodologies.

Lastly, we conclude this Review by emphasizing the necessity and importance of infrastructure for computer software and data set handling for the next generation of CG modeling. As models and derivation strategies increase in complexity, infrastructure for software and data becomes much more crucial. Since the naissance of modern computer simulations for molecules,^483^ computer software has been inseparable from molecular models. Given the *ad hoc* nature of many existing CG models, CG modeling software should be amenable to new feature implementation and easy dissemination to facilitate both access and utility to the general scientific community. Currently, there are several options that satisfy these criteria with different features and capabilities according to their objectives: VOTCA^185^ (BI, IBI; https://github.com/votca), MAGIC^484,485^ (IMC; http://bitbucket.org/magic-su/magic-3), BOCS^486^ (g-YBG, iter-YBG; https://github.com/noid-group/BOCS), openMSCG^487^ (MS-CG, iter-MS-CG, MC-CG, REM; https://software.rcc.uchicago.edu/mscg/), and CGNet^59^ (ML-based approaches; https://github.com/coarse-graining/cgnet). Continued development in both CG methodology and software necessitates standardized data sets that are used to validate existing or new CG methods. For example, in the free energy sampling community, alanine dipeptide serves as a standard example to validate new methodologies,^488^ and ultralong atomistic MD trajectories for fast-folding proteins^489−491^ using the ANTON supercomputer by the D. E. Shaw lab^492^ have been extensively employed in kinetics studies such as MSMs. Even though CG modeling tackles a variety of chemical and biological systems across many different scales, there are relatively few data sets that are publicly available in the CG community. Therefore, by taking inspiration from the ML community, future efforts should also aim to achieve community agreement on which data sets are reliable to be shared and standardized in order to benchmark various CG methodologies. Altogether, we expect that a continuous exploration along these directions will push the frontiers of bottom-up CG modeling into the exploration and characterization of increasingly complex molecular systems.
